# Pharmacological Studies on the Role of 5-HT_1__A_ Receptors in Male Sexual Behavior of Wildtype and Serotonin Transporter Knockout Rats

**DOI:** 10.3389/fnbeh.2020.00040

**Published:** 2020-03-31

**Authors:** Diana Carolina Esquivel-Franco, Sietse F. de Boer, Marcel Waldinger, Berend Olivier, Jocelien D. A. Olivier

**Affiliations:** ^1^Groningen Institute for Evolutionary Life Sciences, University of Groningen, Groningen, Netherlands; ^2^Programa de Doctorado en Ciencias Biomédicas, Universidad Nacional Autónoma de México, Mexico City, Mexico; ^3^Instituto de Investigaciones Biomédicas (IIB), Universidad Nacional Autónoma de México, Mexico City, Mexico; ^4^Department of Pharmacology & Physiology, College of Medicine, Drexel University, Philadelphia, PA, United States; ^5^Department of Psychopharmacology, Utrecht Institute for Pharmaceutical Sciences, Science Faculty, Utrecht University, Utrecht, Netherlands; ^6^Department of Psychiatry, School of Medicine, Yale University, New Haven, CT, United States

**Keywords:** serotonin, male sexual behavior, rat, 5-HT_1__A_ receptor, serotonin transporter, 5-HT_1__A_ autoreceptors, 5-HT_1__A_ heteroreceptors

## Abstract

Brain serotonin (5-HT) neurotransmission plays an important role in male sexual behavior and it is well established that activating 5-HT_1__A_ receptors in rats facilitate ejaculatory behavior. However, the relative contribution of 5-HT_1__A_ somatodendritic autoreceptors and heteroreceptors in this pro-sexual behavior is unclear. Moreover, it is unclear whether the contribution of somatodendritic 5-HT_1__A_ autoreceptors and postsynaptic 5-HT_1__A_ heteroreceptors alter when extracellular 5-HT levels are chronically increased. Serotonin transporter knockout (SERT^–/–^) rats exhibit enhanced extracellular 5-HT levels and desensitized 5-HT_1__A_ receptors. These rats model neurochemical changes underlying chronic SSRI-induced sexual dysfunction. We want to determine the role of presynaptic versus postsynaptic 5-HT_1__A_ receptors in the pro-sexual effects of 5-HT_1__A_ receptor agonists in SERT^+/+^ and in SERT^–/–^ rats. Therefore, acute effects of the biased 5-HT_1__A_ receptor agonists F-13714, a preferential 5-HT_1__A_ autoreceptor agonist, or F-15599, a preferential 5-HT_1__A_ heteroreceptor agonist, and S15535 a mixed 5-HT_1__A_ autoreceptor agonist/heteroreceptor antagonist, on male sexual behavior were assessed. A clear and stable genotype effect was found after training where SERT^+/+^ performed sexual behavior at a higher level than SERT^–/–^ rats. Both F-15599 and F-13714 induced pro-sexual activity in SERT^+/+^ and SERT^–/–^ animals. Compared to SERT^+/+^, the F13714-dose-response curve in SERT^–/–^ rats was shifted to the right. SERT^+/+^ and SERT^–/–^ rats responded similar to F15599. Within both SERT^+/+^ and SERT^–/–^ rats the potency of F-13714 was much stronger compared to F-15599. S15535 had no effect on sexual behavior in either genotype. In SERT^+/+^ and SERT^–/–^ rats that were selected on comparable low sexual activity (SERT^+/+^ 3 or less ejaculations and SERT^–/–^ 5 or less ejaculations in 10 weeks) S15535 also did not influence sexual behavior. The two biased compounds with differential effects on 5-HT_1__A_ auto- and hetero-receptors, exerted pro-sexual activity in both SERT^+/+^ and SERT^–/–^ rats. Applying these specific pharmacological tools has not solved whether pre- or post-synaptic 5-HT_1__A_ receptors are involved in pro-sexual activity. Moreover, the inactivity of S15535 in male sexual behavior in either genotype was unexpected. The question is whether the *in vivo* pharmacological profile of the different 5-HT_1__A_ receptor ligands used, is sufficient to differentiate pre- and/or post-synaptic 5-HT_1__A_ receptor contributions in male rat sexual behavior.

## Introduction

The serotonergic system plays an important modulatory role in sexual behavior (Uphouse and Guptarak, 2010; Olivier et al., 2019). This is, for example, illustrated by the effects of chronic SSRI treatment in depressed patients that results in enhanced 5-HT levels often causing sexual dysfunctions like in men delayed ejaculation and libido problems (Segraves and Balon, 2014). Early studies in male rats identified 5-HT_1__A_ receptor (R) agonists like 8-OH-DPAT, the azapirones (e.g., buspirone, ipsapirone, and gepirone) and others (e.g., flesinoxan) as pro-sexual drugs (Ahlenius et al., 1981; Ahlenius and Larsson, 1985; reviewed in: Snoeren et al., 2014). The prototypal 5-HT_1__A_ receptor agonists (±) and (+) – 8-OH-DPAT, potently stimulate male rat sexual behavior; in a certain time frame (e.g., 30 min), the number of ejaculations increases associated with shortened ejaculation latencies and fewer intromissions to reach ejaculation (Hillegaart and Ahlenius, 1998; Uphouse and Guptarak, 2010; Chan et al., 2011). Although (±) 8-OH-DPAT has also 5-HT_7_ R agonistic effects (Thomas et al., 1999), this mechanism cannot explain the pro-sexual effects because other 5-HT_1__A_ receptor agonists without 5-HT_7_ R agonistic activity, also display pro-sexual effects (Snoeren et al., 2014). 5-HT_1__A_ receptors are present as presynaptic inhibitory autoreceptors on soma and dendrites of raphé serotonergic neurons projecting to many forebrain areas (Fernandez-Guasti et al., 1992; Le Poul et al., 1995; Marek, 2010; Altieri et al., 2013). Moreover, 5-HT_1__A_ receptors are also present as post-synaptic heteroreceptors in various brain areas, mainly in the forebrain (Frink et al., 1996; Garcia-Garcia et al., 2017). Systemic acute administration of non-selective 5-HT_1__A_ receptor agonists like (±)-8-OH-DPAT and flesinoxan (activation of pre- and post-synaptic receptors) leads to decreased serotonergic release, but at the same time to activation of post-synaptic 5-HT_1__A_ heteroreceptors (Müller et al., 2007; Lladó-Pelfort et al., 2012). These non-selective 5-HT_1__A_ receptor agonists often display biphasic dose-response curves, and it is suggested that low doses of 8-OH-DPAT preferentially activate autoreceptors, whereas higher doses of 8-OH-DPAT preferentially activate post-synaptic heteroreceptors (De Vry et al., 2004; Depoortère et al., 2019). However, in male sexual behavior 8-OH-DPAT exerts a linear dose-dependent increase in sexual activities, without any evidence for differential effects on 5-HT_1__A_ auto- or heteroreceptors (Mos et al., 1991). The pro-sexual effects of these non-selective 5-HT_1__A_ receptor agonists are therefore not yet explained in terms of pre- or post-synaptic mechanisms. To further explore the role of pre- and post-synaptic 5-HT_1__A_ receptors in male sexual behavior, more recently developed selective and high-affinity 5-HT_1__A_ receptor agonists are useful. These so-called “biased” or “functionally selective” agonists (Newman-Tancredi, 2011; Garcia-Garcia et al., 2014) display selectivity for either pre- or post-synaptic 5-HT_1__A_ receptors. F15599 is a high-affinity, selective 5-HT_1__A_ receptor agonist (Ki = 3.4 nM) for post-synaptic 5-HT_1__A_ heteroreceptors, whereas F13714 (Ki = 0.1nM) is a preferential 5-HT_1__A_ autoreceptor agonist (Koek et al., 2001; de Boer and Newman-Tancredi, 2016; Hazari et al., 2017). In contrast to 8-OH-DPAT, both F15599 (Newman-Tancredi et al., 2009) and F15714 (Assié et al., 2006) are devoid of 5-HT_7_ receptor activity. We studied both compounds in a dose-response study in male rat sexual behavior. Another high-affinity (Ki = 1.8 nM) 5-HT_1__A_ receptor ligand, S-15535 acts *in vivo* as a preferential agonist at presynaptic autoreceptors and as antagonist at post-synaptic 5-HT_1__A_ heteroreceptors (Millan et al., 1993; Carli et al., 1999). This compound is an interesting tool to study in male sexual behavior as it may shed further light on the complex role of 5-HT_1__A_ receptors in male rat sexual behavior.

As mentioned before, chronic SSRI treatment results in enhanced 5-HT levels often causing sexual dysfunctions (Segraves and Balon, 2014). The exact mechanisms for these dysfunctions remain unclear, but are high likely due to alterations in the 5-HT_1__A_ receptor. Male rats lacking the serotonin transporter (SERT^–/–^) display a robust genotype that has a lower basal ejaculatory performance than wildtype rats (SERT^+/+^) or heterozygous serotonin transporter knockout (SERT^+/–^) rats (Chan et al., 2011; Esquivel-Franco et al., 2018). More specific, due to the lack of the serotonin transporter SERT^–/–^ rats have a nine-fold increase in extracellular 5-HT levels (Homberg et al., 2007), decreased number of ejaculations and an increased ejaculation latency (Chan et al., 2011) compared to SERT^+/+^ rats. This genetic animal model has therefore been proposed and used as an animal model of spontaneous or SSRI-induced delayed ejaculation in humans. Chronic SSRI use in men may result in several side-effects including increased ejaculation threshold, resulting in a delayed ejaculation latency or sometimes even absent ejaculation, associated with a reduction in sexual desire (Waldinger et al., 1998; Hirschfeld, 2003; Balon, 2006; Rubio-Casillas et al., 2015). This is believed to be caused by the combination of enhanced 5-HT levels and diminished 5-HT_1__A_ receptor functioning (both pre- and post-synaptic) similar to chronic SSRI-treatment in normal animals (Chan et al., 2011), or short acting SSRIs like dapoxetine in fast ejaculating rats (Clément et al., 2012). Although conflicting findings on the effects of acute and chronic SSRI treatment have been reported, Clément et al. (2012) mention this is explained by distinct pharmacokinetics rather than pharmacodynamic properties as dapoxetine has rapid peak plasma concentrations which delays ejaculation frequencies in men with premature ejaculation. In rats the dapoxetine profile is less clear, although it is suggested by the authors that in faster ejaculating rats dapoxetine seems to delay the ejaculation latency (Clément et al., 2012). In particular this 5-HT_1__A_ receptor desensitization phenomenon is relevant here to further provide more clarity as to the potency of the biased agonists to stimulate sexual behavior. SERT^–/–^ rats have higher extracellular serotonin levels than SERT^+/+^ animals which is comparable to levels after chronic SSRI administration (Homberg et al., 2007). Pharmacological experiments in these rats indicated that rats lacking the SERT have altered 5-HT_1__A_ receptor reactivity; the altered 5-HT_1__A_ receptor functioning is probably not a global phenomenon, but might be limited to some specific subpopulations of 5-HT_1__A_ receptors (not necessarily pre- or post-synaptic), as indicated by changed autonomic responses like core body temperature in SERT^+/+^ and SERT^–/–^ animals. The 5-HT_1__A_ receptor population involved with hypothermia was not sensitive, while the 5-HT_1__A_ receptor population involved with hyperthermia was still sensitive (Homberg et al., 2008; Olivier et al., 2008). Experiments performed in male sexual behavior (Chan et al., 2011) also indicated that likely at least two populations of 5-HT_1__A_ receptors are involved in its expression. However, it is worthwhile to mention that 5-HT_1__A_ receptors can co-localize with 5-HT_7_ receptors in the cell-membrane (Renner et al., 2012). It has been postulated that heterodimerization of these receptors may influence the desensitization of 5-HT_1__A_ autoreceptors caused by SSRIs (Naumenko et al., 2014). In addition, 5-HT_1__A_ receptors are G-protein coupled receptors activating different intracellular signaling pathways, which are brain region specific. Activation of different G-protein cascades may therefore play a role in the activation of 5-HT_1__A_ receptors in specific cellular environments, while having no effect on other subpopulations of the same receptor (Newman-Tancredi, 2011). For performing sexual behavior, activation of one population of 5-HT_1__A_ receptors is needed and we postulated that this pool is desensitized in SERT^–/–^ rats. The pro-sexual effects of 8-OH-DPAT are probably mediated via 5-HT_1__A_ receptors, which are not changed or somewhat less sensitive in SERT^–/–^ rats. This difference makes the SERT^–/–^ rat a further attractive model to test the different 5-HT_1__A_ receptor-modulating drugs, F15599, F13714 and S-15355, as it may provide information on the adaptation of pre- and post-synaptic 5-HT_1__A_ receptors due to chronic high 5-HT levels, which may aid in the treatment of sexual dysfunction caused by SSRI treatment.

Finally, we selected male rats that, after extensive training (Pattij et al., 2005), display a low level of sexual behavior, i.e., low number of ejaculations. Because 5-HT_1__A_ receptor agonists facilitate ejaculation, a too high initial level of the number of ejaculations would probably interact with the pro-sexual effects of these drugs. The purpose of this study was to use functionally selective agonists for either pre- or post-synaptic 5-HT_1__A_ receptors to identify the role of somatodendritic (auto) receptors and post (hetero) receptors in sexual behavior. A second goal was to use SERT^–/–^ rats because they model SSRI-induced delayed ejaculation in humans, and hence may provide insight in the adaptation of specific 5-HT_1__A_ hetero- or auto receptors due to chronic increased extracellular 5-HT levels. Thus, we investigated whether chronic exposure to high 5-HT levels affected the pro-sexual effects of 5-HT_1__A_ agonists, and whether pre- or post-synaptic receptors were differently affected in SERT^–/–^ rats compared to SERT^+/+^ rats. We used F13714, F15599, and S15535 in normal (SERT^+/+^) and SERT^–/–^ rats, and hypothesized that these drugs would have differential effects on sexual behavior and that SERT^–/–^ rats would display desensitized response to these drugs.

## Materials and Methods

### Animals

Wistar rats were bred in our animal facility (University of Groningen, GELIFES) using serotonin transporter (SERT) heterozygous males and females, resulting in male and female SERT wild type (SERT^+/+^), heterozygous (SERT^±^) and homozygous or knock out (SERT^–/–^) rats. On postnatal day 21 pups were weaned and ears were punched for individual recognition and genotyped as reported previously (El Aidy et al., 2017). We used two groups of animals, the first one (normal ejaculating rats) consisting of sixty-three male SERT (SERT^+/+^, *n* = 32), and (SERT^–/–^, *n* = 31) rats and the second one (slow ejaculating rats) of 32 male (16 SERT^+/+^ and 16 SERT^–/–^) rats, all of them of at least 12 weeks old when used for sexual behavior experiments.

Female SERT^±^ and SERT^+/+^ were used as sexual stimulus females (*n* = 120) as SERT^+/+^ and SERT^±^ rats do not differ in basal sexual activity (Snoeren et al., 2010). Rats were housed under reversed dark-light conditions (12 h light:12 h dark, lights off from 8:00 AM to 8 PM). After 6-weekly training tests (30 min/test), male rats were considered sexually trained and classified based on ejaculation frequencies per test. Male rats display, after extensive training, a rather stable sexual phenotype (Pattij et al., 2005; Olivier et al., 2006; Chan et al., 2008). In these experiments, for the normal ejaculating 24 rats were selected (from 14 different dams, a maximum of 3 SERT^+/+^ and/or 3 SERT^–/–^ rats were used from the same litter) that showed a normal ejaculatory phenotype (between 1 and 2 ejaculations per test after training, for the last three sessions) and for the slow ejaculating rats 20 (from 8 dams, a maximum of 5 SERT^+/+^ and/or 2 SERT^–/–^ rats were used from the same litter) that showed a rather low sexual phenotype (between 0 and 1 ejaculation per test after training, for the last three sessions) were selected. We summed all ejaculations per rat for all training weeks in Supplementary Figure 1 (group 1) and 2 (group 2). The most left tail-side of the distribution was selected. Animals were socially housed (2–5 per cage, maximum 4 for males). Cages were enriched with wooden gnawing blocks and nesting material (Envirodri).

Thus, to select normal ejaculating rats 32 SERT^+/+^ and 31 SERT^–/–^ rats were sexually trained for 6 weeks and a total of 12 SERT^+/+^ and 12 SERT^–/–^ rats were selected with a normal average number of ejaculations. For selection of the slow ejaculating rats 16 SERT^+/+^ and 16 SERT^–/–^ rats were sexually trained for 6 weeks and a total of 10 SERT^+/+^ and 10 SERT^–/–^ rats were selected with a normal and low average number of ejaculations (because this enhances the sensitivity of the anticipated improvement in sexual behavior by the 5-HT_1__A_ compounds and to match the control group as much as possible to the knock-out animals). Experiments in the normal ejaculating rats lasted 13 weeks in total (after training), and 4 weeks in total (after training) for sow ejaculating rats. Animals were used only once a week to guarantee sufficient drug washout time. Rats had *ad libitum* access to food and water. This study was carried out in accordance with the principles of the EU Directive 2010/63/EU. All efforts were made to minimize the number of animals and their suffering.

### Female Rats

Female stimulus rats were tubal ligated in order to prevent pregnancies. To perform tubal ligation surgery, females were anesthetized (Isoflurane) and given pain relief (Fynadine, 0.1 mg/100 g) before surgery, and 24 and 48 h after surgery. Females were at least 12 weeks old when surgery was performed, and 2 weeks of recovery were given before they were made intentionally receptive with estradiol (50 μg in 0.1 ml oil, S.C., 36–48 h before the test) before the sexual behavior training tests and experiments. Females were used not more than once in 2 weeks and not more than two times per experimental day.

### Drug Treatment and Behavioral Experiments

For the first experiment in normal ejaculating rats, animals received all dosages of F13714 and F15599 in a crossover-randomized design in order to prevent order effects; after this experiment, S15535 was administered in a randomized design. For the second experiment in slow ejaculating rats, animals were only administered S15535 in a randomized design similar to the first set of animals. As described previously in Olivier et al. (2017), when pharmacological tests are performed, male rats are given a 30-min habituation time in the test boxes right after drug administration via IP injection, before the female rat is introduced. All behavior during the 30-min test is video-recorded after introduction of the female and were also live-scored; the following parameters of the ejaculation series were deduced (Chan et al., 2011): number of ejaculations/test (E), number of mounts (M), number of intromissions (I), latency (s) to first mount (ML), latency (s) to first intromission (IL) and latency (s) to the first ejaculation (EL). After ejaculation, the post ejaculatory latency (PEL(s)) was calculated, using the time from the first ejaculation and the time of the first mount/intromission (whatever occurred first) of the second ejaculation series. Intromission Ratio (IR) was calculated as: IR = (#I/(#I + #M)) × 100%. EL was calculated using the time of the EL from the first ejaculation series minus the intromission latency of the first ejaculation series (EL = EL – IL). These parameters were used to run the statistical analysis.

Because it is important to have comparable pharmacodynamics and kinetics in pharmacological studies, a test of fixed duration has been chosen: 30 min (1800 s). In the cases where drug-treatment had no “effect” on ejaculation and sexual behavior, or few or no animal achieved a first ejaculation it was not possible to perform statistical analyses and for those cases we assigned values of 1800 s (i.e., the maximum test duration) for some latencies (ejaculation, mount and intromission latency), although this is undoubtedly a matter of discussion as we have discussed before (Chan et al., 2011; Olivier et al., 2017). All tables and figures show the results for the first Ejaculation Series only.

### Drugs

F15599 and F13714 (Pierre Fabre Pharmaceuticals, France; Lot # SBR1401003 and # JLM3001201, resp.) and S-15535 (Servier Pharmaceuticals, France; Lot B01JLP061A) were dissolved in NaCl 0.9% (saline) and each solution was freshly prepared on each testing day. All drugs were administered via intraperitoneal (IP) injection 30 min before the test.

### Training (Table 1)

For the normal ejaculating group, rats were sexually trained for 6 times (30 min, once a week). For the slow ejaculating group, rats were sexually trained 10 times (30 min, once a week). The latter animals received extra training due to the extreme low sexual performance to assess and stabilize their basal sexual activity. Rats habituated for 10 min to the testing box right before the training session. After the habituation period a receptive female was introduced in the box and sexual behavior was assessed for 30 min. Non-receptive females were switched for a different receptive female. The training and testing occurred in wooden rectangular (57 cm × 82 cm × 39 cm; glass wall) testing boxes filled with regular bedding material. To stimulate sexual behavior, bedding material was not changed during the training and testing to preserve pheromones of previous rounds and to create a more competitive sexual environment.

**TABLE 1:** Overview of training of the various genotypes and pharmacological experiments in selected male rats.

**Selection**	**# Rats trained**	**# Rats in pharmacological experiments**	**Drug (doses) tested**
Normal ejaculating rats	32 SERT^+/+^	Exp. 1: 12 SERT^+/+^	F15599 (0.01,0.04, 0.16,
	31 SERT^–/–^	12 SERT^–/–^	and 0.64-mg/kg, IP) F13714 (0, 0.0025, 0.01, 0.04, and 0.16-mg/kg, IP)
		Exp. 2: 12 SERT^+/+^	S15535 (0, 0.25, 1, and 4-mg/kg, IP)
		12 SERT^–/–^	

Slow ejaculating rats	16 SERT^+/+^	Exp. 3: 10 SERT^+/+^	S15535 (0, 0.25, 1, and 4-mg/kg, IP)
	16 SERT^–/–^	10 SERT^–/–^	

Only males showing stable normal (1–2 ejaculations, for experiments 1 and 2 and low (0–1 ejaculations for experiment 3) ejaculation levels in the last three tests were used in the pharmacological experiments. For Experiments 1 and 2 (normal ejaculating rats) 24 rats were selected (*N* = 12 per genotype). In Experiment 3 (slow ejaculating rats) 20 animals were selected (10 per genotype). All training sessions and experiments were performed under red light conditions between 10:00 AM and 17:00 PM.

### Pharmacological Experiments (Table 1)

#### Experiment 1 (Normal Ejaculating Rats): F15599 and F13714 Dose Response

Twenty-four normal ejaculating male rats were selected (*N* = 12 per SERT genotype) and were tested in a crossover design. Rats received vehicle (saline), 0.01, 0.04, 0.16, and 0.64-mg/kg F15599 and 0.0025, 0.01, 0.04, and 0.16-mg/kg F13714 via intraperitoneal (IP) administration. Experiments were performed once per week on the same testing day, over 9 weeks and animals and treatment were randomized over the 9 weeks. Although the experiments with these two drugs were performed together, we performed the statistical analysis separately for each compound.

#### Experiment 2 (Normal Ejaculating Rats): S15535 Dose Response

The same 24 animals from experiment one received vehicle (saline), 0.25, 1 and 4-mg/kg S15535, IP in a randomized design. Testing was performed over 4 weeks and always on the same day per week.

#### Experiment 3 (Slow Ejaculating Rats): S15535 Dose Response

10 SERT^+/+^ and 10 SERT^–/–^ rats were selected for low numbers of ejaculation. Rats received vehicle (saline), 0.25, 1, and 4-mg/kg S15535, via IP administration in a randomized design. Testing was performed over 4 weeks and always on the same day per week.

### Statistical Analyses

Differences in baseline ejaculation numbers during the training between genotypes were analyzed using two-way ANOVA for repeated measures, with genotype as between- and time (weeks) as within-subjects factors. Where appropriate, an independent *t*-test was performed. For the F19955, F13714, and S15535 dose-response experiments, a two-way ANOVA for repeated measures was performed with dose as within-subject factor (5 levels) and genotype as between-subject factor (2 levels). Where appropriate one way-ANOVA with LSD *post hoc* was performed. All statistical analyses were performed using the Statistical Package for Social Sciences for Windows version 25 (LEAD technologies, Chicago, United States). Level of significance was set at *p* < 0.05.

## Results

### Sexual Stability

**FIGURE 1:**
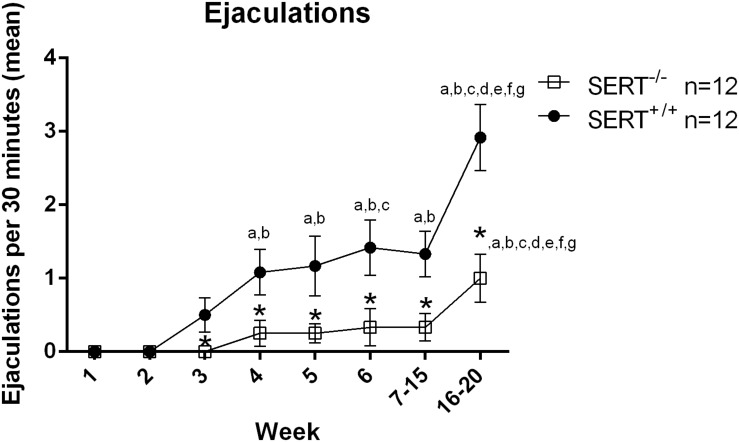
Mean ejaculation frequencies (±SEM) over 6 weeks of training of male Wistar rats of group one (selection for normal ejaculating rats). Added are also the mean ± SEM of the saline data from experiment one (F13714 and F15599) and two (S15535) of group one. a: significantly different (*p* < 0.05) from week 1; b: significantly different (*p* < 0.05) from week 2;c: significantly different (*p* < 0.05) from week 3; d: significantly different (*p* < 0.05) from week 4; e: significantly different (*p* < 0.05) from week 5; f: significantly different (*p* < 0.05) from week 6; g: significantly different (*p* < 0.05) from week 7 to 15; *significantly different (*p* < 0.05) from SERT^+/+^. Detailed statistical analyses are provided in Table 2.

The sexual performance of the selected experimental animal groups that exhibited a normal (1–2 ejaculations) and a low basal ejaculation frequency (0–1 ejaculation) during the six training days was registered and from the 63 male rats sexually trained from the first group and 32 from the second group, only 24 and 20 animals (respectively) that showed stable normal and low sexual performance and ejaculations respectively, were selected to run the pharmacological studies (see Supplementary Figures 1, 2). For the first group (selection for normal ejaculation rats), there was a significant week (time) effect *F*_(__7_._154__)_ = 13.86, *p* < 0.001, a significant week × genotype effect *F*_(__7_._154__)_ = 3.40, *p* < 0.01 and a significant genotype effect (*F*_(__1_,_22__)_ = 23.81, *p* < 0.001). In SERT^+/+^ rats from week 3 onward they performed significant more ejaculations (all *p*-values are < 0.05) compared to the first 2 weeks (Figure 1 and Table 2). In SERT^–/–^ rats only week 16–20 significantly differed (all *p*-values are < 0.05) from all other weeks (Figure 1 and Table 2). SERT^–/–^ rats ejaculated significantly less compared to SERT^+/+^ rats in week 3 (*p* < 0.05), week 4 (*p* < 0.05), week 5 (*p* < 0.05), week 6 (*p* < 0.05), weeks 7–14 (0.05), and weeks 16–20 (*p* < 0.01).

**TABLE 2:** Sexual Behavior performance during training weeks of male SERT^+/+^ and SERT^–/–^ Wistar rats from group 1 (selection of normal ejaculating rats).

**Week**									
**SERT**	**1**	**2**	**3**	**4**	**5**	**6**	**7–15**	**16–20**	**One way ANOVA time effect**
	**Mean ± SEM**	**Mean ± SEM**	**Mean ± SEM**	**Mean ± SEM**	**Mean ± SEM**	**Mean ± SEM**	**Mean ± SEM**	**Mean ± SEM**		
	**A**	**B**	**C**	**D**	**E**	**F**	**G**	**H**		
+/+	0.0 ± 0.0	0.0 ± 0.0	0.50 ± 0.23	1.08 ± 0.31	1.16 ± 0.40	1.41 ± 0.37	1.33 ± 0.30	2.91 ± 0.45	*F*_(__7__.88__)_ = 9.37; *p* < 0.001
				A,B	A,B	A,B,C	A,B	A,B,C,D,E,F,G	
−/−	0.0 ± 0.0	0.0 ± 0.0	0.0 ± 0.0	0.25 ± 0.17	0.25 ± 0.13	0.33 ± 0.25	0.33 ± 0.18	1.00 ± 0.32	*F*_(__7,88__)_ = 3.25; *p* < 0.01
								A,B,C,D,E,F,G	
*t*-test genotype per week	ns	ns	*	*	*	*	*	*	
			*T*_(__1,22__)_ = 2.171, *p* < 0.05	*T*_(__1,22__)_ = 2.311, *p* < 0.05	*T*_(__1,22__)_ = 2.154, *p* < 0.05	*T*_(__1,22__)_ = 2.370, *p* < 0.05	*T*_(__1,22__)_ = 2.760, *p* < 0.05	*T*_(__1,22__)_ = 3.443, *p* < 0.01	
Two-way ANOVA repeated measures	Time (week) effect *F*_(__7,154__)_ = 13.855, *p* < 0.001								
	Time (week) × genotype effect *F*_(__7,154__)_ = 3.396, *p* < 0.01								
	Genotype effect *F*_(__1,22__)_ = 23.807, *p* < 0.001								

**N* = 12/group. A: significantly different from week 1; B: significantly different from week 2; C: significantly different from week 3; D: significantly different from week 4; E: significantly different from week 5; F: significantly different from week 6; G: significantly different from week 7–15; all *p*-values are < 0.05; ns: non significant.*

**FIGURE 2:**
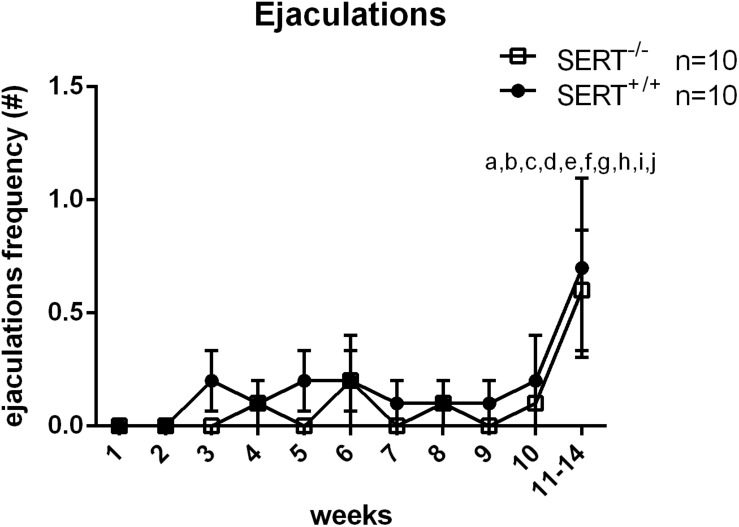
Mean ejaculation frequencies (±SEM) over 10 weeks of training of male Wistar rats of group two (selection for slow ejaculating rats). Added is also the mean ± SEM of the saline data from S15535 experiment (week 11–14). a: significantly different (*p* < 0.05) from week 1; b: significantly different (*p* < 0.05) from week 2; c: significantly different (*p* < 0.05) from week 3; d: significantly different (*p* < 0.05) from week 4; e: significantly different (*p* < 0.05) from week 5; f: significantly different (*p* < 0.05) from week 6; g: significantly different (*p* < 0.05) from week 7; h: significantly different (*p* < 0.05) from week 8; i: significantly different (*p* < 0.05) from week 9; j: significantly different (*p* < 0.05) from week 10; *significantly different (*p* < 0.05) from SERT^+/+^. Detailed statistical analyses are provided in Table 3.

For the second group of animals trained (for selection of slow ejaculating rats), there was a significant difference in weeks of training (*F*_(__10_,_180__)_ = 3.453, *p* < 0.001). In week 11–14, SERT^+/+^ and SERT^–/–^ rats had significant more ejaculations compared to all other weeks (all *p*-values < 0.01). No significant differences in time × genotype, and genotype effects were found during the training weeks (Figure 2 and Table 3).

**TABLE 3:** Sexual Behavior performance during training weeks of male SERT^+/+^ and SERT^–/–^Wistar rats from group 2 (selection for slow ejaculating rats).

**Week**												
**SERT**	**1**	**2**	**3**	**4**	**5**	**6**	**7**	**8**	**9**	**10**	**11–14**	**One Way ANOVA Time effect**
	**Mean ± SEM**	**Mean ± SEM**	**Mean ± SEM**	**Mean ± SEM**	**Mean ± SEM**	**Mean ± SEM**	**Mean ± SEM**	**Mean ± SEM**	**Mean ± SEM**	**Mean ± SEM**	**Mean ± SEM**		
	**A**	**B**	**C**	**D**	**E**	**F**	**G**	**H**	**I**	**J**	**K**		
+/+	0.0 ± 0.0	0.0 ± 0.0	0.20 ± 0.13	0.10 ± 0.10	0.20 ± 0.13	0.20 ± 0.20	0.10 ± 0.10	0.10 ± 0.10	0.10 ± 0.10	0.20 ± 0.20	0.70 ± 0.39	
−/−	0.0 ± 0.0	0.0 ± 0.0	0.0 ± 0.0	0.09 ± 0.09	0.0 ± 0.0	0.18 ± 0.12	0.0 ± 0.0	0.09 ± 0.09	0.0 ± 0.0	0.09 ± 0.09	0.54 ± 0.24	
											A,B,C,D,E,F,G,H,I,J	
*t*-test genotype per week	NA	NA	NA	NA	NA	NA	NA	NA	NA	NA	NA	*F*_(__10_,_220__)_ = 3.418, *p* < 0.001
Two-way ANOVA repeated measure	Time (week) effect *F*_(__10,180__)_ = 3.453, *p* < 0.001											
	No time (week) × Genotype effect *F*_(__10,180__)_ = 0.147, n.s.											
	No genotype effect *F*_(__1,18__)_ = 1.976, n.s.											

**N* = 10 and 11, respectively. A: significantly different from week 1; B: significantly different from week 2; C: significantly different from week 3; D: significantly different from week 4; E: significantly different from week 5; F: significantly different from week 6; G: significantly different from week 7; H: significantly different from week 8; I: significantly different from week 9; J: significantly different week 10; all *p*-values are < 0.01; NA: non applicable.*

We included in Figure 1 the saline data gathered in the pharmacological experiments performed on animals in group one (selection for normal ejaculating rats). The saline data obtained for all animals in weeks 7–15 (Exp. 1) were comparable to the last training data, but the saline data from the last (S15535) experiment (Exp. 2) (during week 16–20) showed significantly higher values. This “enhanced” baseline level of sexual behavior made us decide (because of possible ceiling effects) to repeat the S15535 experiment in rats with very low levels of sexual ejaculation activity (group two, Exp. 3: data shown in Table 3). In Figure 2 we also included the saline data gathered in the S15535 dose-response experiment (Exp. 3) of the second group (week 11–14). Again, an enhanced baseline level of sexual behavior was found in the saline treated animals during the S-155355 treatment weeks.

### Dose-Response of F15599 (Figure 3 and Supplementary Table 1)

**FIGURE 3:**
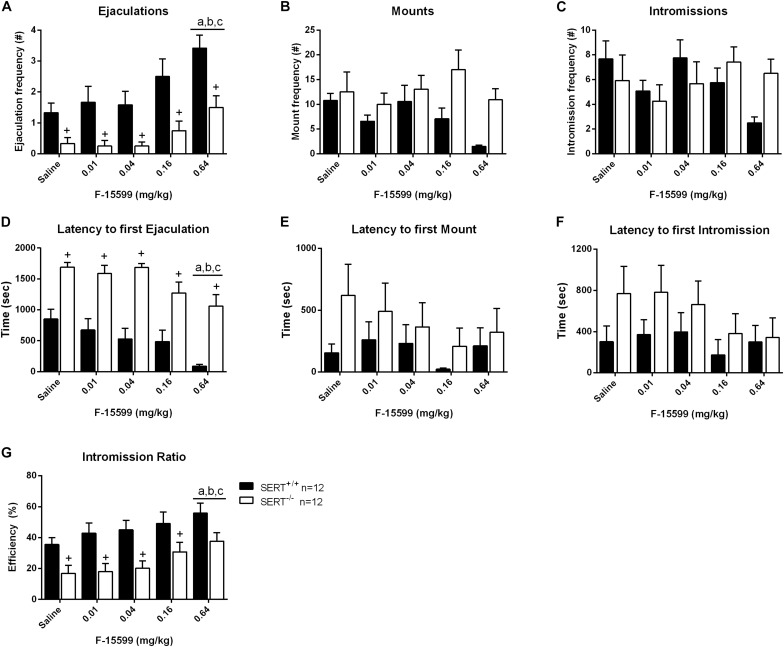
Sexual behavior of male rats treated with 0, 0.01, 0.04, 0.16, or 0.64 mg/kg, IP of F-15599. The number and latency of ejaculations per 30 min **(A,D)**, number and latency of Mounts **(B,E)**, number and latency of Intromissions **(C,F)** and Intromission Ratio **(G)** of the first Ejaculation Series are displayed. Detailed statistical analyses are provided in Supplementary Table 1. a: significant difference (*p* < 0.05) compared to saline group, b: significant difference (*p* < 0.05) compared to 0.01mg/kg group, c: significant difference (*p* < 0.05) compared to 0.04/mg/kg group. +Significant difference between SERT^+/+^ and SERT^–/–^ (*p* < 0.05).

In the dose-response experiment significant dose (*F*_(__4_,_88__)_ = 8.75; *p* < 0.001) and genotype (*F*_(__1_,_22__)_ = 22.278; *p* < 0.001) effects, but no interactions, were found for the number of ejaculations. Comparable significances were found for ejaculation latencies and intromission ratios (see Supplementary Table 1 for statistics of all behavioral parameters). Further analysis revealed that the lowest and intermediate doses of F15599 (0, 0.01, 0.04, and 0.16 mg/kg) had no significant effects on sexual behavior in either genotype (Figure 3 and Supplementary Table 1). Compared to saline (*p* < 0.001), 0.01 (*p* < 0.01), and 0.04 (*p* < 0.001) mg/kg doses of F-15599, the highest dose (0.64 mg/kg) significantly increased the ejaculation frequency. Moreover, ejaculation latencies were significant shorter in 0.64 mg/kg F15599 compared to saline (*p* < 0.01), 0.01 mg/kg (*p* < 0.05), and 0.04 mg/kg (*p* < 0.01) F15599 (Figure 3 and Supplementary Table 1) in both SERT^+/+^ and SERT^–/–^ animals; the 0.64 mg/kg F15599 dose also significantly increased the efficiency of the animals to ejaculate (IR; *p* < 0.05; Figure 3 and Supplementary Table 1) compared to saline (*p* < 0.01), 0.01 mg/kg (*p* < 0.05), and 0.04 mg/kg (*p* < 0.05) of F15599.

A significant decrease in the number of ejaculations of SERT^–/–^ rats was found compared to SERT^+/+^ rats in the saline treatment (*p* < 0.05), and in the 0.01 mg/kg (*p* < 0.05), 0.04 mg/kg (*p* < 0.05), 0.16 mg/kg (*p* < 0.05), and 0.64 mg/kg (*p* < 0.05) F15599 treatment. Similarly, an increase in ejaculation latency was found for SERT^–/–^ rats compared to SERT^+/+^ rats in saline treatment (*p* < 0.001), and in 0.01 mg/kg (*p* < 0.01), 0.04 mg/kg (*p* < 0.001), 0.16 mg/kg (*p* < 0.05), and 0.64 mg/kg (*p* < 0.001) F15599 treatment. For the intromission ratio, a significant decrease was found for SERT^–/–^ rats compared to SERT^+/+^ rats in saline treatment (*p* < 0.05), and in the 0.01 mg/kg (*p* < 0.01), 0.04 mg/kg (*p* < 0.05), and 0.16 mg/kg (*p* < 0.05) F15599 treatment.

### Dose-Response of F13714 (Figure 4 and Supplementary Table 2)

**FIGURE 4:**
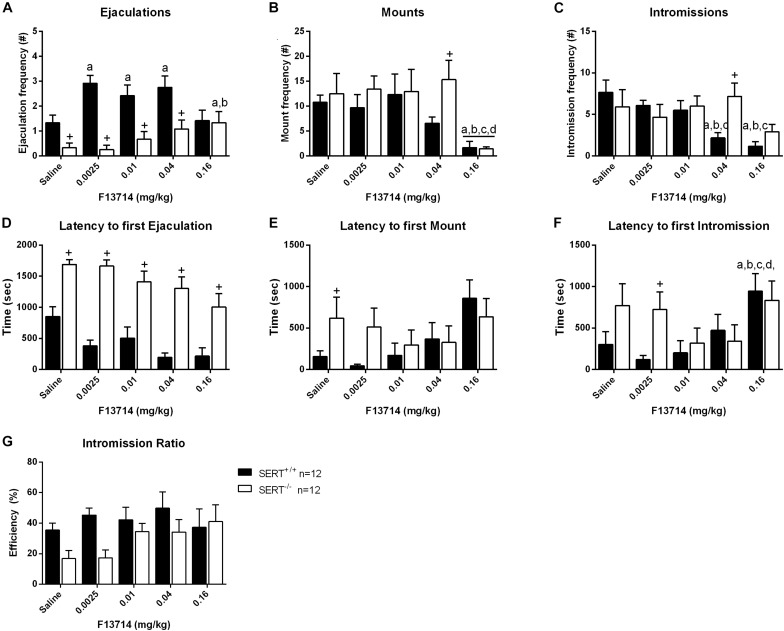
Sexual behavior of male rats treated with 0, 0.0025, 0.01, 0.04, or 0.16 mg/kg F13714. The number and latency of ejaculations per 30 min **(A,D)**, number and latency of Mounts **(B,E)**, number and latency of Intromissions **(C,F)**, and Intromission Ratio **(G)** of the first Ejaculation Series are provided. Detailed statistical analyses are displayed in Supplementary Table 2. a: significant difference (*p* < 0.05) compared to saline group, b: significant difference (*p* < 0.05) compared to 0.0025mg/kg group, c: significant difference (*p* < 0.05) compared to 0.01/mg/kg group. +Significant difference between SERT^+/+^ and SERT^–/–^ (*p* < 0.05).

Overall, F13714 induced pro-sexual effects in both genotypes, although the dose-effect curves for both genotypes differed considerably (Figure 4 and Supplementary Table 2). Considering ejaculations, significant dose (*F*_(__4_,_88__)_ = 3.287, *p* < 0.05), genotype (*F*_(__1_,_22__)_ = 20.649, *p* < 0.001), and genotype × dose interactions (*F*_(__4_,_88__)_ = 4.810, *p* < 0.01) were found. Comparable significances were found for ejaculation latencies (see Supplementary Table 2 for statistics of all behavioral parameters). In SERT^+/+^ rats, F13714 stimulated sexual behavior significantly, illustrated (compared to saline) in the increase in ejaculation frequencies at 0.0025 mg/kg (*p* < 0.01), 0.01 mg/kg (tendency; *p* = 0.06), and 0.04 mg/kg (*p* < 0.05) mg/kg F13714. In the SERT^–/–^ rats, pro-sexual effects were observed only at the highest dose (0.16 mg/kg) compared to saline (*p* < 0.05) and 0.025 mg/kg (*p* < 0.05) of F13714. Although the ejaculation latency was decreased at this high dose for both genotypes, the difference was not statistically significant. The number of mounts was equally decreased in SERT^+/+^ and SERT^–/–^ rats at 0.16 mg/kg F13714 compared to saline (*p* < 0.01), 0.025 mg/kg (*p* < 0.01), 0.01 mg/kg (*p* < 0.001) and 0.04 mg/kg (*p* < 0.05) F13714. In SERT^+/+^, but not in SERT^–/–^, rats, the intromission latency was enhanced at the highest dose (*F*_(__4_,_55__)_ = 4.203, *p* < 0.01). The intromission latency at the highest dose (0.16 mg/kg) of F13714 was significant longer compared to saline (*p* < 0.01), 0.0025 mg/kg (*p* < 0.001), 0.01 mg/kg (*p* < 0.01), and 0.04 mg/kg (*p* < 0.05) F13714. Lastly, the number of intromissions was significantly decreased in SERT^+/+^ rats only (*F*_(__4_,_55__)_ = 8.194; *p* < 0.001). Intromissions were significant reduced in animals treated with 0.16 mg/kg F13714 compared to saline (*p* < 0.001), 0.0025 mg/kg (*p* < 0.01) and 0.01 mg/kg (*p* < 0.01) F13714 treated SERT^+/+^ rats. In addition, 0.04 mg/kg F13714 treated SERT^+/+^ rats had a significant reduced number of intromissions compared to those treated with saline (*p* < 0.001), 0.0025 mg/kg (*p* < 0.01), and 0.01 mg/kg (*p* < 0.05) F13714.

SERT^–/–^ rats had significant lower ejaculation frequencies compared to SERT^+/+^ rats after treatment with saline (*p* < 0.05), and after treatment with 0.0025 mg/kg (*p* < 0.01), 0.01 mg/kg (*p* < 0.01), and 0.04 mg/kg (*p* < 0.01) F13714. For mounts, only at a dose of 0.04 mg/kg F13714 SERT^–/–^ rats showed a significant higher mount frequency (*p* < 0.01) compared with SERT^+/+^ rats. At the same dose SERT^–/–^ rats also showed a higher intromission frequency compared to SERT^+/+^ rats. For latency to the first ejaculation a significant increase was found for SERT^–/–^ rats compared to SERT^+/+^ rats for all doses (all *p*-values < 0.01). The latency to the first mount was significantly higher for SERT^–/–^ rats compared to SERT^+/+^ after saline treatment (*p* < 0.05) and the latency to the first intromission was also significantly higher for SERT^–/–^ rats compared to SERT^+/+^ at 0.0025 mg/kg F13714 (*p* < 0.05).

### Dose-Response of S15535 (Figures 5, 6 and Supplementary Tables 3, 4)

**FIGURE 5:**
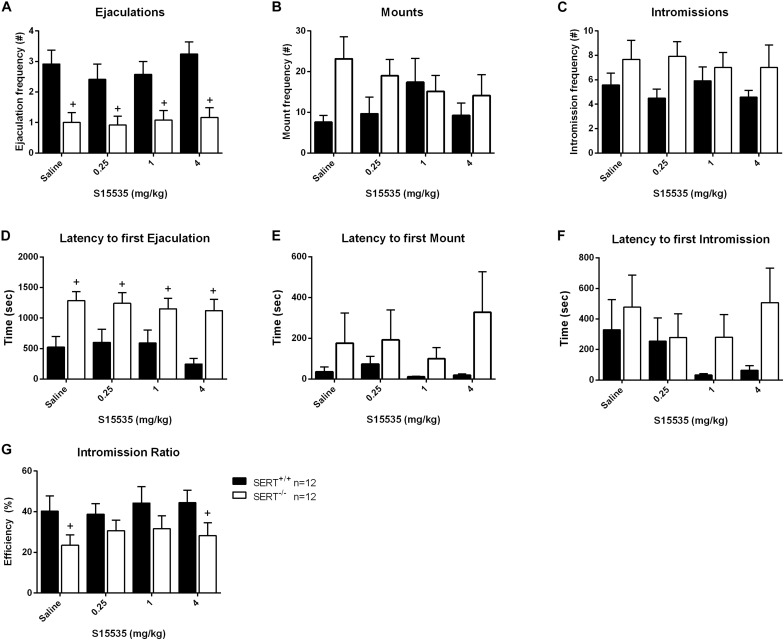
Sexual behavior of male rats from normal ejaculating rats treated with 0, 0.25, 1, or 4 mg/kg S15535. The number and latency of ejaculations per 30 min **(A,D)**, number and latency of Mounts **(B,E)**, number and latency of Intromissions **(C,F)**, post-ejaculatory interval **(G)**, and Intromission Ratio **(H)** of the first Ejaculation Series are provided. Detailed statistical analyses are displayed in Supplementary Table 3. +Significant difference between SERT^+/+^ and SERT^–/–^ groups.

**FIGURE 6:**
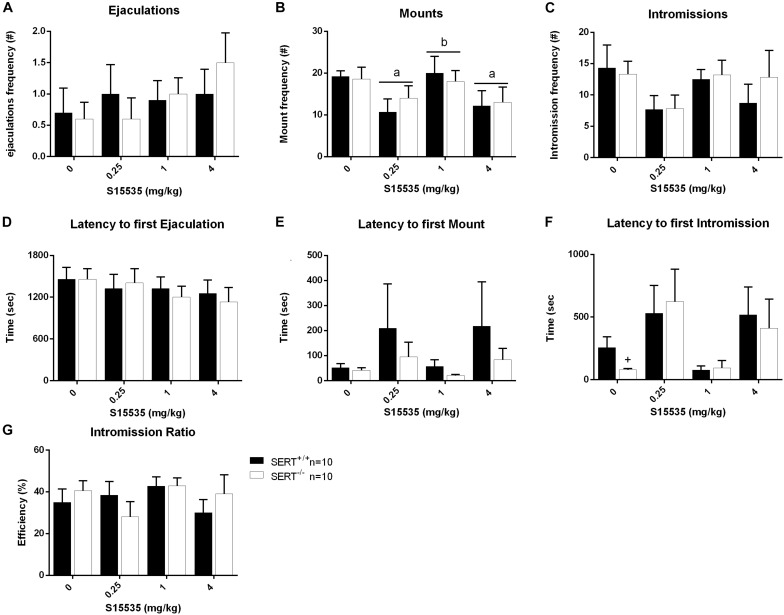
Sexual behavior of male rats from slow ejaculating treated with 0, 0.25, 1, or 4 mg/kg S15535. The number and latency of ejaculations per 30 min **(A,D)**, number and latency of Mounts **(B,E)**, number and latency of Intromissions **(C,F)** and Intromission Ratio **(G)** of the first Ejaculation Series are given. Detailed statistical analyses are shown in Supplementary Table 4. a: significant difference (*p* < 0.05) compared to saline group, c: significant difference (*p* < 0.05) compared to 1/mg/kg group. +Significant difference between SERT^+/+^ and SERT^–/–^ (*p* < 0.05).

S15535 (0.25, 1, and 4-mg/kg) had no significant effects on sexual behavior in SERT^+/+^ and SERT^–/–^ (Figures 5, 6) compared to saline in either group of animals (Exp. 2 and 3). In the normal ejaculating rats (Exp. 2), a significant genotype effect for ejaculation frequencies was found (*F*_(__1_,_22__)_ = 21.167, *p* < 0.001; Figure 5A). SERT^+/+^ rats had significant higher ejaculation frequencies after treatment with saline (*p* < 0.001), 0.25 mg/kg (*p* < 0.001), 1 mg/kg (*p* < 0.05), and 4 mg/kg (*p* < 0.001) S15535 in comparison with SERT^–/–^ rats. Similar effects were found for the ejaculation latency (*F*_(__1_,_22__)_ = 25.627, *p* < 0.001; Figure 5D and Supplementary Table 3) where there was an increase for SERT^–/–^ versus SERT^+/+^ animals after saline treatment (*p* < 0.001), and after treatment with 0.25 mg/kg (*p* < 0.05), 1 mg/kg (*p* < 0.05), and 4 mg/kg (*p* < 0.001) S15535, and to some extent in the intromission ration, although this was only significant in the saline treated (*p* < 0.05) and 4 mg/kg (*p* < 0.05) S155355 treated group.

In the slow ejaculating rats (Exp. 3), no significant differences were found in the majority of parameters measured, although a significant dose effect was found for the number of mounts (*F*_(__3_,_54__)_ = 3.077, *p* < 0.05). Analysis revealed a significant reduction in the number of mounts between saline and 0.025 mg/kg (*p* < 0.05), between saline and 4 mg/kg S-155355 (*p* < 0.05) and between 0.025 and 1 mg/kg S-155355 (*p* < 0.05). In addition, a genotype effect was found in the latency to the first intromission (*F*_(__1_,_18__)_ = 5.786, *p* < 0.05). Compared to SERT^+/+^, SERT^–/–^ displayed a shorter latency to the first intromission (*p* < 0.05; see Figure 6 and Supplementary Table 4).

### Comparison Between F15599 and F13714 (Supplementary Figure 3)

A fit curve plot for SERT^+/+^ and SERT^–/–^ rats on a log scale (see Supplementary Figure 3) was made where data were normalized against the saline treated group. The ED_50_ was calculated for SERT^+/+^ (F15599, ED_50_ = 0.21 mg/kg; F13714, ED_50_ = 0.0065 mg/kg) and SERT^–/–^ (F15599, ED_50_ = 0.165 mg/kg; F13714, ED_50_ = 0.00178 mg/kg) and illustrated that F13714 was more potent compared to F15599 in both SERT^+/+^ and SERT^–/–^ rats. The curved fit plot also showed that SERT^–/–^ rats were sensitive to both compounds, as they were able to increase the percentage of ejaculations compared with the saline treated group.

## Discussion

In the present study, after extensive training of the two genotypes studied (SERT^+/+^ and SERT^–/–^), animals showed two different but stable sexual phenotypes, confirming earlier findings (Chan et al., 2011) where male SERT^+/+^ rats performed sexual behavior at a higher level than SERT^–/–^ rats. Permanent changes in serotonergic processes in the central nervous system by removing the SERT protein from conception on (Chan et al., 2011; Olivier et al., 2011), apparently leads to permanent changes in overt male sexual behavior in rats. The male rat sexual behavior paradigm used in the present studies has been developed over the last decades (Pattij et al., 2005; Chan et al., 2008; Olivier et al., 2011), specifically to test the effects of psychoactive drugs, including antidepressants (Waldinger and Olivier, 2005; Chan et al., 2010; Heijkoop et al., 2018). The paradigm is able to distinguish acute effects of drugs like the pro-sexual effects of 5-HT_1__A_ receptor agonists (Pattij et al., 2005), but also the chronic inhibitory effects of SSRI antidepressants (Chan et al., 2010, 2011; Bijlsma et al., 2014). Pro-sexual effects of drugs in male rat sexual behavior are reflected in the speed of onset of sexual activity toward a newly introduced female in behavioral estrus; reflected in a shorter interval to reach ejaculation (Andersson and Larsson, 1994), including reduced number of mounts and intromissions to reach ejaculation and enhanced number of ejaculations over a certain test period (in our case 30 min). Reduction of sexual behavior, e.g., by chronic antidepressants (Chan et al., 2010; Bijlsma et al., 2014) has reversed effects. This chronic SSRI (antidepressant)-induced profile of reduced male sexual behavior is comparable to the sexual behavior of SERT^–/–^ rats and supports the hypothesis that male SERT^–/–^ rats are modeling the sexual effects of chronic SSRI administration (Chan et al., 2011; Olivier et al., 2011). Several studies on SERT-genotypes in sexual behavior have been performed in at least three different labs (Utrecht, Groningen, Netherlands) and Hefei (China: Geng et al., 2019). In all three independent studies male SERT^–/–^ rats display a significantly lower level of sexual behavior than SERT^+/+^ rats (Olivier et al., 2011; Geng et al., 2019; Olivier and Olivier, 2019). It can be suggested that full absence of the SERT reduces the level of each individual rat’s sexual behavior. It can be speculated that the resulting sexual phenotype of a SERT^–/–^ rat may be derived from a certain basic sexual behavior that in some way is permanently inhibited when the SERT is absent from conception on. This can also be illustrated by the comparable ejaculation curves (# ejaculations after training) for both genotypes of which the SERT^–/–^ rats are shifted to the left compared to the SERT^+/+^ rats (Olivier and Olivier, 2019). The typical distribution patterns of the # of ejaculations (or the 1st ejaculation latency times) in a large cohort of SERT-genotypes (Olivier and Olivier, 2019) supplies us with the possibility to behaviorally match animals with a certain genotype, e.g., only high versus low sexually performing animals, as we did previously in the study of tramadol effects on male sexual behavior (Esquivel-Franco et al., 2018). In the present experiments we intentionally created (Exp. 2) two groups of SERT^+/+^ and SER^–/–^ rats with low (not statistically different) levels of sexual behavior in order to circumvent possible interference of high versus low rates of behavior.

Two biased 5-HT_1__A_ receptor agonists, the preferential 5-HT_1__A_ auto-receptor agonist F13714 (Assié et al., 2006; Becker et al., 2016) and the preferential 5-HT_1__A_ heteroreceptor agonist F15599 (Newman-Tancredi et al., 2009; Becker et al., 2016) were tested in SERT^+/+^ and SERT^–/–^ rats. Both compounds induced pro-sexual activity in SERT^+/+^ and SERT^–/–^ rats (for overview see Table 4). F13714 is considerably more potent than F15599 in eliciting the pro-sexual effects, but the similarity of the response of both compounds on male sexual behavior suggests that both compounds share comparable mechanisms of action in evoking sexual behavior. This may point to an autoreceptor-mediated effect. Unfortunately, full dose-response curves of this pro-sexual effect were not available for both compounds making definite conclusions impossible. In F13714-treated SERT^–/–^ rats the dose-response curve of pro-sexual activity was shifted to the right compared to SERT^+/+^ rats, but this was not the case in F15599 treated rats where the sexual inhibiting doses were comparable in both genotypes. 5-HT_1__A_ receptor stimulation by “non-selective” (with regard to pre- and post-synaptic receptors) 5-HT_1__A_ receptor agonists like 8-OH-DPAT, flesinoxan, buspirone, ipsapirone, and others (Olivier et al., 1999) have pro-sexual effects in wildtype rats (Snoeren et al., 2014 for review), but no studies were performed before where the specific contributions of 5-HT_1__A_ auto-receptors or 5-HT_1__A_ heteroreceptors (or both) are investigated. S15535, an auto-receptor selective 5-HT_1__A_ receptor agonist and heteroreceptor-selective 5-HT_1__A_ receptor antagonist, did not have any effects on male sexual behavior of SERT^+/+^ and SERT^–/–^ rats, neither in normal ejaculating (on average 1–2 ejaculations/30 min; group 1) nor in slow ejaculating (0–1 ejaculations/30 min; group 2) rats. We conclude that S15535 behaves as a “silent” 5-HT_1__A_ receptor ligand in male rat sexual behavior.

**TABLE 4:** Overview of ejaculatory responses to 5-HT_1__A_ receptor agonists in SERT^+/+^ and SERT^–/–^-/ rats.

	**F15599**	***F*13714**	***S*15535**	***S*15535**
			***Normal ejaculating rats***	***Slow ejaculating rats***
SERT^+/+^ rats	↑	↑ at lower dose	No effect	No effect
SERT^–/–^ rats	↑	↑ at higher dose	No effect	No effect

*↑: increased ejaculation frequency.*

The prototypal 5-HT_1__A_ receptor agonist (±) or (+)-8-OH-DPAT, a non-selective auto-receptor and heteroreceptor agonist (Larsson et al., 1990), has strong and dose-dependent pro-sexual effects (Mos et al., 1991; Chan et al., 2011; Snoeren et al., 2014). This pro-sexual effect can be fully antagonized by the 5-HT_1__A_ receptor antagonist WAY100,635, a behaviorally silent compound (de Jong and Neumann, 2015). In male SERT^–/–^ rats (Chan et al., 2011) 8-OH-DPAT had pro-sexual effects, although (like the biased agonist F13714 in the present study) the dose-response curve was shifted to the right compared to SERT^+/+^ rats. The lack of any behavioral effect of S15535 in either SERT^+/+^ or SERT^–/–^ rats is rather puzzling. Apparently, 5-HT_1__A_ receptor antagonistic activity on 5-HT_1__A_ heteroreceptors in SERT^–/–^ rats did not cause inhibition of male sexual behavior like WAY100,635 treatment (Chan et al., 2011). The stimulating effect of F13714 and F15599 in male sexual behavior in both SERT^+/+^ and SERT^–/–^ rats is also quite puzzling, because it makes explanations in term of pre- or post-synaptic 5-HT_1__A_ receptor mechanisms involved, troublesome. However, it remains possible that the preferential post-synaptic 5-HT_1__A_ receptor agonist F15599, at higher doses (like in this experiment) also displays some presynaptic autoreceptor agonistic activity. In that case F15599 does not appear the specific tool to selectively activate post-synaptic 5-HT_1__A_ heteroreceptors.

How do the sexual data obtained with these three serotonergic ligands compare to their effects in other behavioral systems? The research group of De Boer (de Boer and Newman-Tancredi, 2016) has tested these (and other) ligands extensively in male rat models of offensive aggression in Wildtype Groningen (WTG) rats. In male rat offensive aggression (de Boer et al., 1999, 2000) 8-OH-DPAT potently and dose-dependently reduced offensive aggression but also induces strong sedative-like behaviors. Because 5-HT_1__A_ receptor agonists induce a so-called serotonin-5-HT_1__A_ syndrome, characterized by Lower Lip Retraction (LLR), Forepaw Treading (FPT), and Flat Body Posture (FBP), it is not completely clear whether this sedative-like activity is similar to these serotonergic behaviors. These anti-aggressive and other effects of 8-OH-DPAT can be fully antagonized by WAY100,635 (de Boer et al., 1999, 2000), a silent antagonist in offensive aggression. F13714, F15599, and S15535 all reduce offensive aggression (de Boer and Newman-Tancredi, 2016). Both F13714 and F15599 induce a serotonergic-5-HT_1__A_ syndrome in rats (Newman-Tancredi et al., 2009; Assié et al., 2010; Jastrzȩbska-Wiȩsek et al., 2018). S15535 does not induce the serotonergic-5-HT_1__A_ syndrome at all (de Boer and Newman-Tancredi, 2016; Jastrzȩbska-Wiȩsek et al., 2018) and also has no sedative-like activity in offensive aggression (de Boer et al., 2000). WAY100,635 antagonized the anti-aggressive action of S15535, F15599, and F13714 (de Boer and Newman-Tancredi, 2016).

If the mechanisms of action of the three 5-HT_1__A_ ligands as extensively investigated by various research groups are true, mechanistic interpretations of the behavioral effects found in male sexual behavior are rather difficult to make. Serotonergic 5-HT_1__A_ auto-receptors in the raphe nuclei are generally considered as, upon activation, leading to inhibition of cell firing and consequently a decrease of serotonin release. Subsequently, all post-synaptic 5-HT (hetero) receptors (including 5-HT_1__A_ heteroreceptors) receive diminished or no stimulation by serotonin and depending on the coupling of the post-synaptic receptor to different transduction mechanisms the neuron involved will be activated or inhibited. Serotonin is also known to crosstalk with non-serotonergic systems which may exert effects on (sexual) behavior as well (e.g., Blier, 2001). In case of a non-selective 5-HT_1__A_ receptor agonist like 8-OH-DPAT, next to its inhibiting action on the serotonergic neuron, direct 5-HT_1__A_ heteroreceptor stimulation still occurs leading to post-synaptically mediated effects, like the serotonergic-5-HT_1__A_ behavioral syndrome (Berendsen et al., 1990; Jastrzȩbska-Wiȩsek et al., 2018). In the case of F13714, a relatively selective (compared to heteroreceptor) 5-HT_1__A_ auto-receptor agonist (Assié et al., 2006) potently facilitated sexual activity in male SERT^+/+^ rats suggesting that pro-sexual activity is related to activation of 5-HT_1__A_ auto-receptors. The relatively selective 5-HT_1__A_ heteroreceptor agonist F15599 also facilitated male sexual activity in SERT^+/+^ rats. The difference in potency (factor 256 difference) to obtain the pro-sexual activity (at the lowest effective dose) can possibly be explained by the difference of the *in vitro* and *in vivo* affinity and efficacy of both compounds on 5-HT_1__A_ receptors (Assié et al., 2010; Newman-Tancredi, 2011; Jastrzȩbska-Wiȩsek et al., 2018). This might be taken as suggestive that both compounds exert pro-sexual activity via activation of 5-HT_1__A_ auto-receptors. Strangely enough, both compounds also activate the serotonergic-5-HT_1__A_ syndrome (Newman-Tancredi, 2011; Becker et al., 2016). The 5-HT_1__A_ auto-receptor agonist S15535 does not induce pro-sexual behavior, neither in normal nor in sexually slow ejaculating rats. Whether blocking of post-synaptic 5-HT_1__A_ heteroreceptors antagonizes the expected pro-sexual effect of the auto-receptor stimulation is rather difficult to envisage. This would assume a rather high level of basal activity of 5-HT_1__A_ heteroreceptors involved in sexual behavior. Interestingly, Pattij et al. (2005) showed that slow, normal and rapid ejaculating rats showed increased ejaculations after treatment with 8-OH-DPAT; however, when rats were re-tested 1 week after this 5-HT_1__A_ receptor agonist administration all phenotypes returned to ejaculatory behavior levels found before the 8-OH-DPAT treatments. In the present study we found that during the weeks where treatment with S155355 were administered, the saline groups (and thus baseline levels) showed significant higher ejaculation frequencies compared to the ejaculation frequencies during the training weeks. This might suggest that pro-sexual effects due to 5-HT_1__A_ receptor agonist can be long-lasting, most likely due to alterations in the 5-HT_1__A_ receptors. Further research is warranted to investigate how long this effect would persist and whether it is, 1 week after all treatments with 5-HT_1__A_ receptor agonists, and without saline treatment, still present.

SERT^–/–^ rats, a model of permanently changed serotonergic activity in the brain (Homberg et al., 2007) and associated with an altered sexual phenotype (Chan et al., 2011) may be helpful in explaining the behavioral effects found for the three compounds. Chan et al. (2011) have found that 8-OH-DPAT has pro-sexual effects in male SERT^–/–^ rats, although the dose-response curve has been shifted to the right compared to SERT^+/+^ rats. Remarkably, WAY100,635, a non-selective 5-HT_1__A_ receptor antagonist and without any behavioral effects in SERT^+/+^ males, was (dose-dependently) inhibitory in SERT^–/–^ rats. WAY100,635 was able to completely antagonize the pro-sexual effects of 8-OHDPAT in SERT^+/+^ rats but only partially in SERT^–/–^ rats (Chan et al., 2011). We concluded from these data that complete absence of SERT molecules had led to alterations in 5-HT_1__A_ receptor functioning, hypothesizing that one pool of 5-HT_1__A_ receptors mediates pro-sexual effects of 5-HT_1__A_ receptor stimulation and is not (de)sensitized, whereas another pool of 5-HT_1__A_ receptors, mediating the inhibitory effects of antagonized 5-HT_1__A_ receptors seems sensitized in the SERT^–/–^ rats. The hypothesis of two differentially regulated 5-HT_1__A_ receptor pools in SERT^–/–^ rats has also been found in autonomic regulation of body temperature and stress (Olivier et al., 2008). The findings with F15599 and F13714 in the SERT^–/–^ rats cannot be explained in terms of action on different 5-HT_1__A_ receptor pools. If any, both compounds seem to activate the pool mediating the pro-sexual effects. The 5-HT_1__A_ heteroreceptor antagonistic effects of S15535 do not lead to inhibition of male sexual behavior in the better performing (normal ejaculating) SERT^–/–^ rats, as was the case for WAY100,635 in the Chan et al. (2011) study.

Our expectation that biased 5-HT_1__A_ receptor agonists and a mixed 5-HT_1__A_ presynaptic receptor agonist and post-synaptic antagonist might help to reveal the potential contribution of these different 5-HT_1__A_ receptors was too optimistic. The mechanisms of action of the respective molecules are probably to complex, especially *in vivo* in complicated networks, where 5-HT_1__A_ receptors interact with various other neurotransmitter systems in the modulation of male sexual behavior.

## Conclusion

The data collected with the pharmacological experiments show that selective (preferential) pre- and postsynaptic 5-HT_1__A_ receptor agonists possess pro-sexual effects in SERT^+/+^ and SERT^–/–^, although the response is diminished in SERT^–/–^ animals, most likely due to desensitization of 5-HT_1__A_ receptors. The pharmacological experiment with S15535 compared with previous experiments performed in aggression lacked any sexual behavioral effect. Further experiments are needed to explore whether separate neurobiological substrates at the 5-HT_1__A_ receptors level exist.

## Data Availability Statement

The raw data supporting the conclusions of this article will be made available by the authors, without undue reservation, to any qualified researcher.

## Ethics Statement

This study was carried out in accordance with the principles of the EU Directive 2010/63/EU.

## Author Contributions

DE-F, BO, and JO contributed with conception and design of the work. DE-F carried out all the experimental work, data collection and analysis, and draft work. DE-F, BO, SB, and JO contributed to the interpretation of the data and results, made sure all parts of the work were appropriately investigated and resolved. DE-F, BO, SB, MW, and JO contributed on revising critically the intellectual content, accountability and accuracy of the work, and gave approval for the publication of the content.

## Conflict of Interest

The authors declare that the research was conducted in the absence of any commercial or financial relationships that could be construed as a potential conflict of interest.
