# Supplementary figures and images for: Pharmacological Studies on the Role of 5-HT1A Receptors in Male Sexual Behavior of Wildtype and Serotonin Transporter Knockout Rats
Source: Front Behav Neurosci. 2020 Mar 31;14:40. doi: 10.3389/fnbeh.2020.00040 (PMC7136541; doi:10.3389/fnbeh.2020.00040)

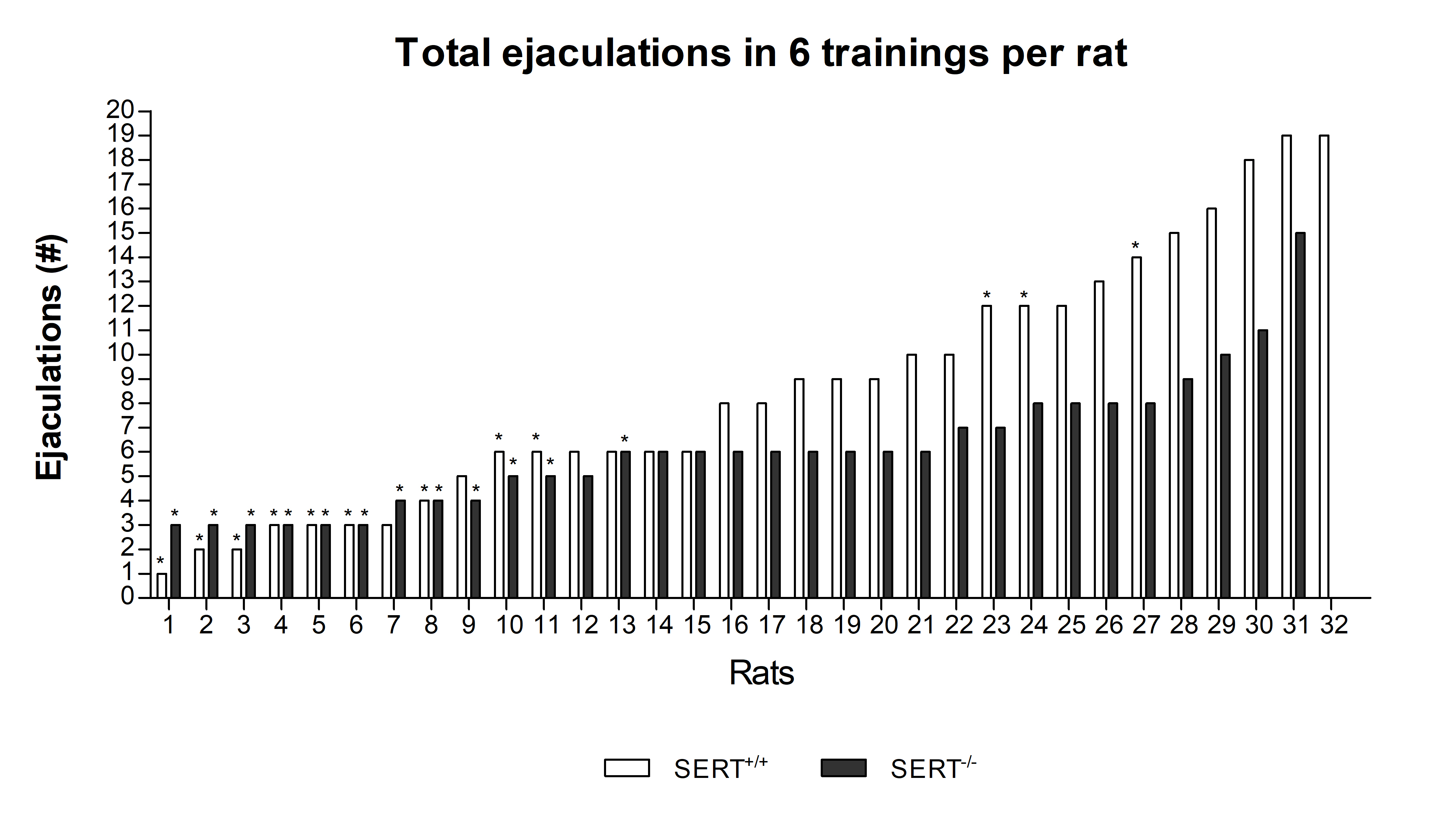

Supplement: Supplementary file 1 [file Image_1.JPEG]

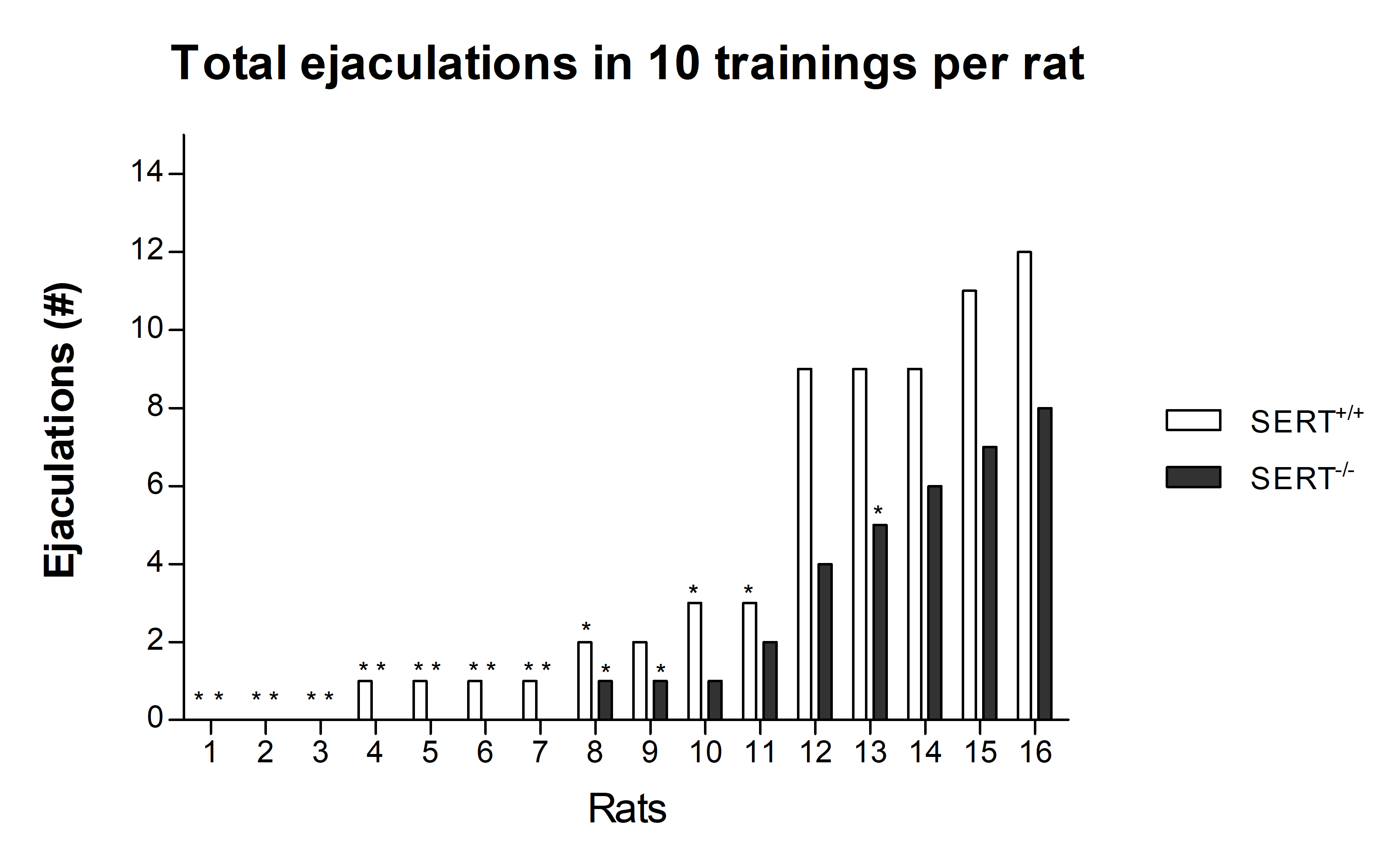

Supplement: Supplementary file 2 [file Image_2.JPEG]

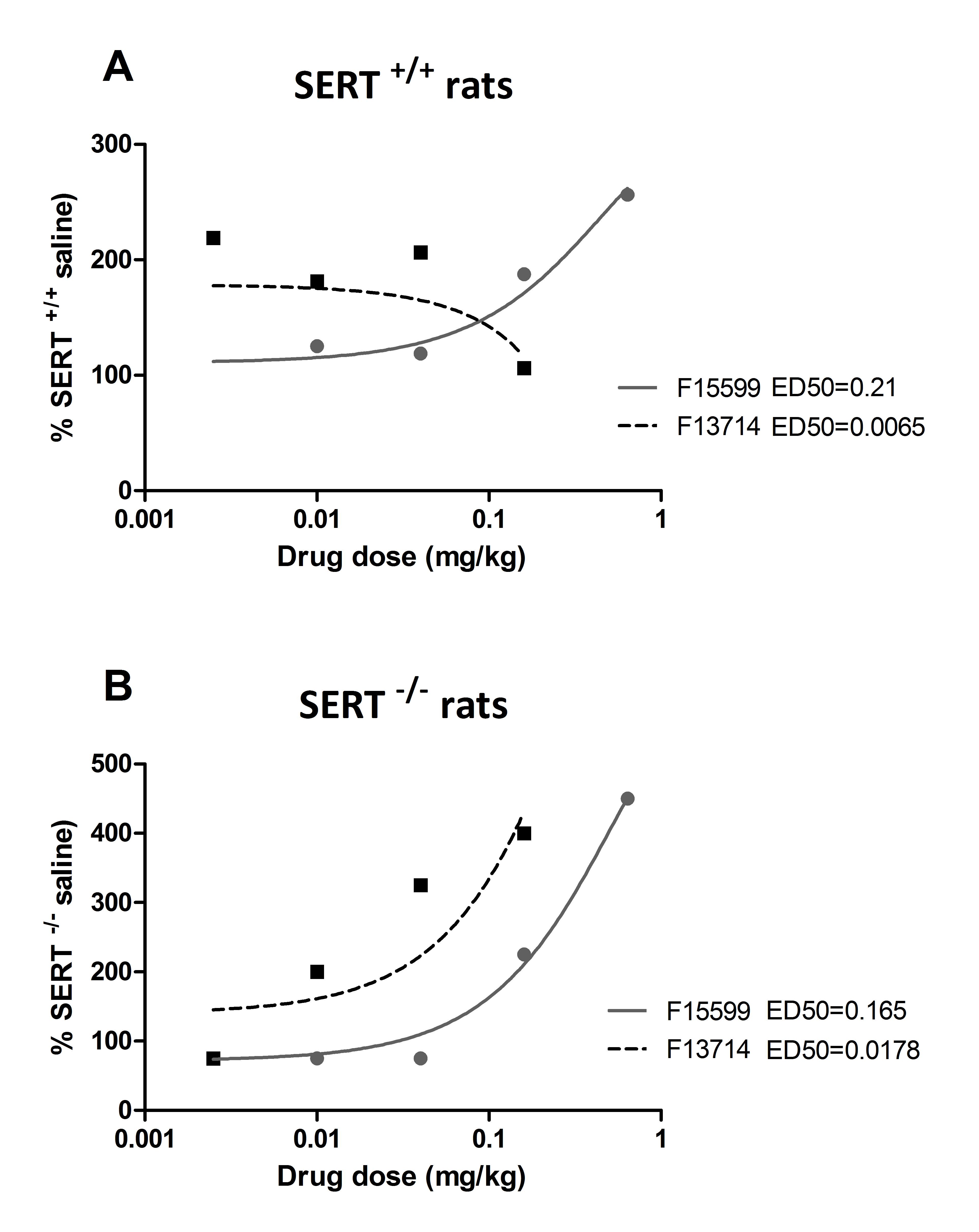

Supplement: Supplementary file 3 [file Image_3.JPEG]
