# Supplementary material for: Pharmacological Studies on the Role of 5-HT1A Receptors in Male Sexual Behavior of Wildtype and Serotonin Transporter Knockout Rats
Source: Front Behav Neurosci. 2020 Mar 31;14:40. doi: 10.3389/fnbeh.2020.00040 (PMC7136541; doi:10.3389/fnbeh.2020.00040)
Supplement: Supplementary file 4 [file Table_1.DOCX]

**Supplementary table 1: Effects of F15599 on Sexual Behavior of male SERT^+/+^ and SERT^-/-^ Wistar rats. N=12/group**

| Dose of F-15599, mg/kg | | | 0 mg/kg  **A** | | | | 0.01 mg/kg  **B** | | | | 0.04 mg/kg  **C** | | | | 0.16 mg/kg  **D** | | 0.64 mg/kg  E | | One Way ANOVA  Dose effect |
| --- | --- | --- | --- | --- | --- | --- | --- | --- | --- | --- | --- | --- | --- | --- | --- | --- | --- | --- | --- |
| Parameters measured | | | Mean ±SEM | | | | Mean ±SEM | | | | Mean ±SEM | | | | Mean ±SEM | | Mean ±SEM | |  |
| *# E SERT +/+*  *SERT -/-*  One-way ANOVA between genotype per dose | | | 1.3±0.30  0.3±0.18  *****  F(_1,22_)= 7.615, p<0.05 | | | | 1.6±0.51  0.25±0.17  *****  F(_1,22_)= 6.807, p<0.05 | | | | 1.58±0.43  0.25±0.136  *****  F(_1,22_)= 8.638, p<0.05 | | | | 2.50±0.57.7.5  0±0.30  *****  F(_1,22_)= 7.317, p<0.05 | | 3.41±0.41  1.50±0.37  **A,B,C**  *****  F(_1,22_)= 11.569; p<0.05 | | F(_4,115_)=5.286, p<0.001 |
| 2-WAY ANOVA repeated measures | | | Dose effect F(_4,88_)=8.747, p<0.001  No Dose*Genotype effect F(_4,88_)=0.594, n.s.  Genotype effect F(_1,22_)=22.278, p<0.001 | | | | | | | | | | | | | | | | |
| *# M 1^st^ series*  *SERT +/+*  *SERT -/-*  One-way ANOVA between genotype per dose | | | 10.75±1.457  12.50±4.061  **NA** | | | | 6.53±1.22  10.00±2.25  **NA** | | | | 10.58±3.23  13.08±2.77  **NA** | | | | 7.08±2.15  17.00±3.97  **NA** | | 1.50±0.26  10.92±2.26  **NA** | | **NA** |
| 2-WAY ANOVA repeated measures | | | Dose effect F(_4,88_)=5.334, p<0.001  No Dose*Genotype effect F(_4,88_)=2.030, n.s.  No Genotype effect F(_1,22_)=2.962, n.s. | | | | | | | | | | | | | | | | |
| *# I 1^st^ series*  *SERT +/+*  *SERT -/-*  One-way ANOVA between genotype per dose | | | 7.66±1.46  5.91±2.07  **NA** | | | 5.08±0.84  4.25±1.32  **NA** | | | | 7.75±1.45  5.66±1.76  **NA** | | | | 5.75±1.16  7.41±1.22  **NA** | | | 2.50±0.46  6.50±1.14  **NA** | **NA** | |
| 2-WAY ANOVA repeated measures | | | No Dose effect F(_4,88_)=1.707, n.s  No Dose*Genotype effect F(_4,88_)=2.114, n.s.  No Genotype effect F(_1,22_)=0.856, n.s | | | | | | | | | | | | | | | | |
| *Latency 1^st^ E (s) SERT +/+*  *SERT -/-*  One-way ANOVA between genotype per dose | | | 853.3±157.3  1689.4±75.8  *****  F(_1,22_)= 22.922, p<0.001 | | | | 673.8±184.6  1589±131.2  *****  F(_1,22_)= 16.335, p<0.01 | | | | 528.6±173.0  1686±62.3  *****  F(_1,22_)= 39.601, p<0.001 | | | | 487.3±183.6  1272±177.2  *****  F(_1,22_)= 9.466, p<0.05 | | 88.67±29.8  1063±184.9  **A,B,C**  *****  F(_1,22_)= 27.033, p<0.001 | | F(_4,115_)= 3.010; p<0.05 |
| 2-WAY ANOVA repeated measures | | | Dose effect F(_4,88_)=7.446, p<0.001  No Dose*Genotype effect F(_4,88_)=0.525, n.s.  Genotype effect F(_1,22_)=77.110, p<0.001 | | | | | | | | | | | | | | | | |
| *Latency 1^st^ M (s) SERT +/+*  *SERT -/-*  One-way ANOVA between genotype per dose | | | 155.5±71.6  619.6±251.7  **NA** | | | | 260.0±146.7  491.3±228.4  **NA** | | | | 232.2±150.7  365.1±195.6  **NA** | | | | 23.8±8.8  208.0±147.8  **NA** | | 211.9±146.8  322.3±192.9  **NA** | | **NA** |
| 2-WAY ANOVA repeated measures | | | No Dose effect F(_4,88_)=1.260, n.s.  No Dose*Genotype effect F(_4,88_)=1.260, n.s.  No Genotype effect F(_1,22_)=1.906, n.s. | | | | | | | | | | | | | | | | |
| *Latency 1^st^ I (s)*  *SERT +/+*  *SERT -/-*  One-way ANOVA between genotype per dose | | | 302.6±152.4  770.1±262.5  **NA** | | | | 371.3±145.3  782.6±259.4  **NA** | | | | 397.1±186.1  663.7±226.4  **NA** | | | | 382.1±148.2  252.4±193.2  **NA** | | 300.2±159.2  342.8±191.0  **NA** | | **NA** |
| 2-WAY ANOVA repeated measures | | | No Dose effect F(_4,88_)=1.418, n.s.  No Dose*Genotype effect F(_4,88_)=0.532, n.s.  No Genotype effect F(_1,22_)=2.252, n.s. | | | | | | | | | | | | | | | | |
| *IR_1_ SERT +/+*  *SERT -/-*  One-way ANOVA between genotype per dose | | | 35.5±4.4  16.8±5.1  *****  F(_1,22_)= 7.501, p<0.05 | | | | 42.8±6.6  18.0±5.2  *****  F(_1,22_)= 8.580, p<0.01 | | | | 45.0±6.2  20.2±4.7  *****  F(_1,22_)= 9.943, p<0.05 | | | | 49.0±7.4  30.7±6.1  *****  F(_1,22_)= 3.608, p<0.05 | | 55.9±6.4  37.6±5.6  **A,B,C**  **ns** | | F(_4,115_)= 3.111,p<0.05 |
| 2-WAY ANOVA repeated measures | | | Dose effect F(_4,88_)=4.566, p<0.01  No Dose*Genotype effect F(_4,88_)=0.205, n.s.  Genotype effect F(_1,22_)=19.573, p<0.001 | | | | | | | | | | | | | | | | |
|  | |  | | |  | | | |  | | | |  | | |  |  |  |  |
|  | |  | | |  | | | |  | | | |  | | |  |  |  |  |
|  | |  | | |  | | | |  | | | |  | | |  |  |  |  |
|  |  | | |  | | | |  | | | |  |  |  |  |  |  |  |  |
|  |  | | |  |  |  |  |  |  |  |  |  |  |  |  |  |  |  |  |

A: significant difference compared to saline group, B: significant difference compared to 0.01mg/kg group, C: significant difference compared to 0.04/mg/kg group, p values set at <0.05 (for specific p values go to results sections). *: significant difference between SERT^+/+^ and SERT^-/-^ (p<0.05).

**Supplementary table 2: Effects of F13714 on Sexual Behavior of male SERT^+/+^ and SERT^-/-^ Wistar rats. N=12/group**

| Dose of F-13714, mg/kg | | 0 mg/kg  **A** | | | 0.0025 mg/kg  **B** | | 0.01 mg/kg  **C** | | | 0.04 mg/kg  **D** | | 0.16 mg/kg  E | | One Way ANOVA  Dose effect | |
| --- | --- | --- | --- | --- | --- | --- | --- | --- | --- | --- | --- | --- | --- | --- | --- |
|  |  | | |  | |  | | |  | |  |  |  |  |  |
| Parameters measured | | Mean ±SEM | | | Mean ±SEM | | Mean ±SEM | | | Mean ±SEM | | Mean ±SEM | |  | |
| *# E SERT +/+*  *SERT -/-*  One-way ANOVA between genotype per dose | | 1.33±0.30  0.33±0.18  *****  F(_1,22_)=7.615, p<0.05 | | | 2.91±0.31  **A**  0.25±0.17  *****  F(_1,22_)=54.680, p<0.01 | | 2.41±0.43  **A**  0.66±0.30  *****  F(_1,22_)=10.156, p<0.01 | | | 2.75±0.46  **A**  1.08±0.35  *****  F(_1,22_)=8.118, p<0.01 | | 1.41±0.41  1.33±0.44  **A,B**  **ns** | | F(_4,55_)= 3.607, p<0.05  F(_4,55_)= 2.230, p=0.078 | |
| 2-WAY ANOVA repeated measures | | Dose effect F(_4,88_)=3.287, p<0.05  Dose*Genotype effect F(_4,88_)=4.810, p<0.01  Genotype effect F(_1,22_)=20.649, p<0.001 | | | | | | | | | | | | | |
| *# M 1^st^ series*  *SERT +/+*  *SERT -/-*  One-way ANOVA between genotype per dose | | 10.7±1.45  12.5±4.06  **ns** | | | 9.6±2.68  13.4±2.63  **ns** | | 12.3±4.11  12.9±4.44  **ns** | | | 6.5±1.22  15.3±3.88  *****  F(_1,22_)=11.642, p<0.01 | | 1.6±1.23  1.4±0.4345  **A,B,C,D**  **ns** | | F(_4,115_)=4.476, p<0.01 | |
| 2-WAY ANOVA repeated measures | | Dose effect F(_4,88_)=2.285, p=0.06  No Dose*Genotype effect F(_4,88_)=1.294, n.s.  Genotype effect F(_1,22_)=6.943, p<0.05 | | | | | | | | | | | | | |
| *# I 1^st^ series*  *SERT +/+*  *SERT -/-*  One-way ANOVA between genotype per dose | | 7.6±1.46  5.9±2.07  **ns** | | | 6.0±0.60  4.6±1.53  **ns** | | 5.5±1.17  6.0±1.22  **ns** | | | 2.1±0.62  **A,B,C**  7.16±1.61  *****  F(_1,22_)=8.305, p<0.01 | | 1.1±0.56  **A,B,C**  2.9±0.88  **ns** | | F(_4,55_)= 8.194; p<0.001  F(_4,55_)= 1.128; n.s. | |
| 2-WAY ANOVA repeated measures | | Dose effect F(_4,88_)=5.308, p<0.01  Dose*Genotype effect F(_4,88_)=3.128, p<0.05  No Genotype effect F(_1,22_)=0.510, n.s. | | | | | | | | | | | | | |
| *Latency 1^st^ E (s) SERT +/+*  *SERT -/-*  One-way ANOVA between genotype per dose | | 853.3±157.3  1689.4±75.8  *****  F(_1,22_)=22.922, p<0.001 | | | 379.3±91.56  1664±97.59  *****  F(_1,22_)=92.148, p<0.001 | | 504.4±178.2  1411±169.8  *****  F(_1,22_)=13.568, p<0.01 | | | 196.3±68.59  1303±186.2  *****  F(_1,22_)=31.127, p<0.001 | | 215.3±133.4  1004±219.3  *****  F(_1,22_)=9.429, p<0.01 | | F(_4,115_)= 1.293; n.s. | |
| 2-WAY ANOVA repeated measures | | Dose effect F(_4,88_)=7.604, p<0.001  Dose*Genotype effect F(_4,88_)=1.253, p<0.05  Genotype effect F(_1,22_)=62.797, p<0.001 | | | | | | | | | | | | | |
| *Latency 1^st^ M (s) SERT +/+*  *SERT -/-*  One-way ANOVA between genotype per dose | | 155.5±71.6  619.6±251.7  *****  F(_1,22_)=3.144, p<0.05 | | | 44.17±19.21  512.1±227.0  **ns** | | 170.8±148.3  296.4±182.36  **ns** | | | 369.0±195.7  327.2±198.8  **ns** | | 858.3±221.2  635.5±221.2  **ns** | | F(_4,115_)= 2.315, n.s. | |
| 2-WAY ANOVA repeated measures | | Dose effect F(_4,88_)=3.160, p<0.05  No Dose*Genotype effect F(_4,88_)=1.79, n.s.  Genotype effect F(_1,22_)=22.47, p<0.001 | | | | | | | | | | | | | |
| *Latency 1^st^ I (s)*  *SERT +/+*  *SERT -/-*  One-way ANOVA between genotype per dose | | 302.6±152.4  770.1±262.5  **ns** | | | 120.4±50.65  722.86±211.3  *****  F(_1,22_)=7.682, p<0.05 | | 201.8±146.0  319.7±181.0  **ns** | | | 472.2±192.2  342.6±197.0  **ns** | | 945.9±209.2  **A,B,C,D**  832.5±234.0  **ns** | | F(_4,115_)= 4.203, p<0.01  F(_4,115_)= 1.266, n.s. | |
| 2-WAY ANOVA repeated measures | | Dose effect F(_4,88_)=4.628, p<0.001  Dose*Genotype effect F(_4,88_)=2.298, p<0.05  No Genotype effect F(_1,22_)=1.029, n.s. | | | | | | | | | | | | | |
| *IR_1_ SERT +/+*  *SERT -/-*  One-way ANOVA between genotype per dose | | 35.5±4.4  16.8±5.1  **NA** | | | 45.3±4.47  17.3±5.19  **NA** | | 42.2±8.22  34.4±5.47  **NA** | | | 49.8±10.64  34.1±8.11  **NA** | | 37.3±11.99  41.0±11.00  **NA** | | **NA** | |
| 2-WAY ANOVA repeated measures | | No Dose effect F(_4,88_)=1.514, n.s.  No Dose*Genotype effect F(_4,88_)=1.291, n.s.  No Genotype effect F(_1,22_)=1.029, n.s. | | | | | | | | | | | | | |
|  |  | | |  | |  | | |  | |  |  |  |  |  |
|  | | |  | | | | |  | | | | |  | |  |

A: significant difference compared to saline group, B: significant difference compared to 0.0025mg/kg group, C: significant difference compared to 0.01/mg/kg group; D: significant difference compared to 0.04/mg/kg group p values set at <0.05 (for specific p values go to results sections). *: significant difference between SERT^+/+^ and SERT^-/-^ (p<0.05).

**Supplementary table 3: Effects of S15535 on Sexual Behavior of male SERT^+/+^ and SERT^-/-^ Wistar rats of normal ejaculating rats. N=12/group**

| Dose of S-15535 mg/kg | 0 mg/kg | 0.25 mg/kg | 1 mg/kg | .4 mg/kg | One Way ANOVA  Dose effect |
| --- | --- | --- | --- | --- | --- |
| Parameters measured | Mean ±SEM | Mean ±SEM | Mean ±SEM | Mean ±SEM |  |
| *# E SERT +/+*  *SERT -/-*  One-way ANOVA between genotype per dose | 3.2±0.39  1.1±0.32  *****  F(_1,22_)=11.851, p<0.001 | 2.4±0.49  0.9±0.28  *****  F(_1,22_)=6.776, p<0.001 | 2.9±0.45  1.0±0.32  *****  F(_1,22_)=6.069, p<0.05 | 2.5±0.41  1.0±0.31  *****  F(_1,22_)=11.380, p<0.001 | **NA** |
| 2-WAY ANOVA repeated measures | No Dose effect F(_3,66_)=0.616, n.s.  No Dose*Genotype effect F(_4,3,66_)=0.360, n.s.  Genotype effect F(_1,22_)=21.167, p<0.001 | | | | |
| *# M 1^st^ series*  *SERT +/+*  *SERT -/-*  One-way ANOVA between genotype per dose | 9.2±3.03  14.1±5.12  **NA** | 9.6±4.05  19.0±3.99  **NA** | 7.5±1.65  23.1±5.43  **NA** | 17.4±5.86  15.1±3.92  **NA** | **NA** |
| 2-WAY ANOVA repeated measures | No Dose effect F(_3,66_)=0.439, n.s.  No Dose*Genotype effect F(_3,66_)=1.575, n.s.  No Genotype effect F(_1,22_)=4.45, n.s. | | | | |
| *# I 1^st^ series*  *SERT +/+*  *SERT -/-*  One-way ANOVA between genotype per dose | 4.5±0.54  7.0±1.8  **NA** | 4.5±0.73  7.9±1.20  **NA** | 5.5±0.97  7.6±1.56  **NA** | 5.9±1.14  7.0±1.24  **NA** | **NA** |
| 2-WAY ANOVA repeated measures | No Dose effect F(_3,66_)=0.827, n.s.  No Dose*Genotype effect F(_3,66_)=0.525, n.s.  Genotype effect F(_1,22_)=3.03, p<0.05 | | | | |
| *Latency 1^st^ E (s) SERT +/+*  *SERT -/-*  One-way ANOVA between genotype per dose | 246.7±94.03  1122±185.5  *****  F(_1,22_)=11.051, p<0.001 | 602.6±213.0  1243±172.5  *****  F(_1,22_)=5.462, p<0.05 | 523.7±175.8  1288±148.1  *****  F(_1,22_)=4.218, p<0.05 | 592.5±213.1  1153±170.3  *****  F(_1,22_)=17.732, p<0.001 | **NA** |
| 2-WAY ANOVA repeated measures | No Dose effect F(_3,66_)=0.869, n.s.  No Dose*Genotype effect F(_3,66_)=0.346, n.s.  Genotype effect F(_1,22_)=25.627, p<0.001 | | | | |
| *Latency 1^st^ M (s) SERT +/+*  *SERT -/-*  One-way ANOVA between genotype per dose | 18.4±6.85  328.3±198.6  **NA** | 73.5±38.28  192.3±147.0  **NA** | 35.2±23.27  176.3±147.8  **NA** | 11.2±3.13  99.5±55.39  **NA** | **NA** |
| 2-WAY ANOVA repeated measures | No Dose effect F(_3,66_)=0.982, n.s.  No Dose*Genotype effect F(_3,66_)=0.988, n.s.  No Genotype effect F(_1,22_)=1.864, n.s. | | | | |
| *Latency 1^st^ I (s)*  *SERT +/+*  *SERT -/-*  One-way ANOVA between genotype per dose | 63.5±31.13  507.3±226.0  **NA** | 254.2±153.3  278.8±155.2  **NA** | 328.3±198.8  477.7±210.0  **NA** | 33.1±8.30  280.6±147.4  **NA** | **NA** |
| 2-WAY ANOVA repeated measures | No Dose effect F(_3,66_)=1.308, n.s.  No Dose*Genotype effect F(_3,66_)=1.008, n.s.  No Genotype effect F(_1,22_)=1.674, n.s. | | | | |
|  |  |  |  |  |  |
| *IR_1_ SERT +/+*  *SERT -/-*  One-way ANOVA between genotype per dose | 44.4±6.19  28.2±6.39  *****  F(_1,22_)=3.440, p<0.05 | 38.7±5.20  30.6±5.16  **ns** | 40.3±7.51  23.5±5.08  **ns** | 44.2±8.0  31.6±6.3  *****  F(_1,22_)=3.294, p<0.05 | **NA** |
| 2-WAY ANOVA repeated measures | No Dose effect F(_3,66_)=0.375, n.s.  No Dose*Genotype effect F(_3,66_)=0.228, n.s.  Genotype effect F(_1,22_)=6.648, p<0.05 | | | | |

*: significant difference between SERT^+/+^ and SERT^-/-^ (p<0.05).

**Supplementary table 4: Effects of S15535 on Sexual Behavior of male SERT^+/+^ and SERT^-/-^ Wistar rats of slow ejaculating rats. N=10 and N=11 respectively**

| Dose of S-15535 mg/kg | 0 mg/kg  **A** | 0.25 mg/kg  **B** | 1 mg/kg  **C** | .4 mg/kg  **D** | One Way ANOVA  Dose effect |
| --- | --- | --- | --- | --- | --- |
| Parameters measured | Mean ±SEM | Mean ±SEM | Mean ±SEM | Mean ±SEM |  |
| *# E SERT +/+*  *SERT -/-*  One-way ANOVA between genotype per dose | 0.70±0.39  0.60±0.26  **NA** | 1.00±0.47  0.60±0.33  **NA** | 0.90±0.31  1.00±0.25  **NA** | 1.00±0.39  1.50±0.47  **NA** | **NA** |
| 2-WAY ANOVA repeated measures | No Dose effect F(_3,54_)=0.619, n.s.  No Dose*Genotype effect F(_3,54_)=0.561, n.s.  No Genotype effect F(_1,18_)=0.005, n.s. | | | | |
| *# M SERT +/+*  *SERT -/-*  One-way ANOVA between genotype per dose | 19.20±1.34  18.60±2.86  **NA** | 10.70±3.11  14.00±2.95  **NA** | 20.00±4.03  18.00±2.65  **NA** | 12.20±3.60  13.00±3.68  **A,C**  **NA** | F(_3,79_)=2.981, p<0.05 |
| 2-WAY ANOVA repeated measures | Dose effect F(_3,54_)=3.077, p<0.05  No Dose*Genotype effect F(_3,54_)=0.281, n.s.  No Genotype effect F(_1,19_)=0.024, n.s. | | | | |
| *# I SERT +/+*  *SERT -/-*  One-way ANOVA between genotype per dose | 14.30±3.66  13.30±2.05  **NA** | 7.70±2.20  7.80±2.19  **NA** | 12.50±1.56  13.20±2.35  **NA** | 8.70±3.01  12.80±4.28  **NA** | **NA** |
| 2-WAY ANOVA repeated measures | No Dose effect F(_3,4_)=1.942, n.s.  No Dose*Genotype effect F(_3,54_)=0.327, n.s.  No Genotype effect F(_1,18_)=0.207, n.s. | | | | |
| *Latency 1^st^ E (s) SERT +/+*  *SERT -/-*  One-way ANOVA between genotype per dose | 1458±171.3  1459±151.6  **NA** | 1324±203.9  1404±205.8  **NA** | 1324±168.4  1202±154.1  **NA** | 1254±192.8  1133±206.1  **NA** | **NA** |
| 2-WAY ANOVA repeated measures | No Dose effect F(_3,54_)=2.155, n.s.  No Dose*Genotype effect F(_3,54_)=0.531, n.s.  No Genotype effect F(_1,18_)=0.162, n.s. | | | | |
| *Latency 1^st^ M (s) SERT +/+*  *SERT -/-*  One-way ANOVA between genotype per dose | 52.00±15.96  41057±10.55  **NA** | 209.5±176.8  95.16±58.21  **NA** | 56.67±26.87  20.88±4.432  **NA** | 218.2±176.5  83.89±44.97  **NA** | **NA** |
| 2-WAY ANOVA repeated measures | No Dose effect F(_3,54_)=0.335, n.s.  No Dose*Genotype effect F(_3,54_)=1.444, n.s.  No Genotype effect F(_1,18_)=0.489, n.s | | | | |
| *Latency 1^st^ I (s)*  *SERT +/+*  *SERT -/-*  One-way ANOVA between genotype per dose | 256.0±86.47  78.53±10.83  *****  F(_1,18_)=4.149, p<0.05 | 530.8±221.5  623.3±258.2  **ns** | 77.90±31.63  94.60±58.41  **ns** | 517.3±223.0  411.4±231.8  **ns** | **NA** |
| 2-WAY ANOVA repeated measures | No Dose effect F(_3,54_)=1.186, n.s.  No Dose*Genotype effect F(_3,54_)=1.188, n.s.  Genotype effect F(_1,18_)=5.786, p<0.05 | | | | |
| *IR_1_ SERT +/+*  *SERT -/-*  One-way ANOVA between genotype per dose | 34.94±6.42  40.56±4.80  **NA** | 38.44±6.502  28.08±7.17  **NA** | 42.76±4.45  42.94±3.732  **NA** | 30.01±6.26  39.04±9.13  **NA** | **NA** |
| 2-WAY ANOVA repeated measures | No Dose effect F(_3,54_)=0.995, n.s.  No Dose*Genotype effect F(_3,54_)=0.977, n.s.  No Genotype effect F(_1,18_)=0.053, n.s | | | | |

A: significant difference compared to saline group, C: significant difference compared to 1/mg/kg group; p values set at <0.05 (for specific p values go to results sections). *: significant difference between SERT^+/+^ and SERT^-/-^ (p<0.05).
