# Supplementary material for: Pharmacological Studies on the Role of 5-HT1A Receptors in Male Sexual Behavior of Wildtype and Serotonin Transporter Knockout Rats
Source: Front Behav Neurosci. 2020 Mar 31;14:40. doi: 10.3389/fnbeh.2020.00040 (PMC7136541; doi:10.3389/fnbeh.2020.00040)
Supplement: Supplementary file 5 [file Table_2.DOCX]

***Figure legends supplementary figures***

Supplementary Fig 1. Cumulative ejaculations over 6 weeks of training of male Wistar rats of group one (selection for normal ejaculating rats). * rats selected for the pharmacological experiments

Supplementary Fig 2. Cumulative ejaculations over 10 weeks of training of male Wistar rats of group two (selection for low ejaculating rats). * rats selected for the pharmacological experiments

Supplementary Fig 3. Fit curve plot for SERT^+/+^ and SERT^-/-^ rats on a log scale comparing F15599 and F13714
